# Use of metabolomics data analysis to identify fruit quality markers enhanced by the application of an aminopolysaccharide†

**DOI:** 10.1039/d1ra05865g

**Published:** 2021-11-03

**Authors:** El Amerany Fatima, Taourirte Moha, Wahbi Said, Meddich Abdelilah, Rhazi Mohammed

**Affiliations:** Laboratory of Bio-Organic Chemistry and Macromolecular, Faculty of Science and Technology of Marrakech, Department of Chemistry, Cadi Ayyad University PO Box 549 Marrakech 40000 Morocco el.amerany.fatima@gmail.com; Natural Macromolecules Team, Normal Graduate School, Department of Biology, University Cadi Ayyad PO Box 575 Marrakech 40000 Morocco; Laboratory of Agro-Food, Biotechnologies and Valorization of Plant Bioresources, Faculty of Science Semlalia, Department of Biology, Cadi Ayyad University PO Box 2390 Marrakech 40000 Morocco

## Abstract

Chitosan is a biostimulator that has a great effect either on plant physiology, productivity, or fruit quality. However, the metabolic mechanism regulated by chitosan still remains unknown. Untargeted metabolomics analysis, using LC-MS/MS mass spectrometry, was used to investigate fruit quality markers. Thus, this study was focused on the identification of untargeted metabolites of tomato fruits produced under the application of five doses of chitosan at different concentrations (0, 0.25, 0.50, 0.75, and 1 mg ml^−1^) that was extracted from *Parapenaeus longirostris* shrimp shells. The identification was carried out using two ion modes (ESI^−^/ESI^+^), a web application “Metfamily” to analyze signals, and reference libraries. The analysis of data using partial least squares discriminant analysis (PLS-DA) and hierarchical cluster analysis (HCA) showed that chitosan application, especially 0.75 mg ml^−1^, had a clear and remarkable effect regarding the number of metabolite families identified in both ion modes. This treatment has increased the relative abundance of many metabolites that belong to anthocyanins decorated with sugars, terpenoids, phenylpropanoids, acylsugars, glucosinolates, folates, galactolipids, fatty acids, and phospholipids. Thus, these results showed that chitosan application increased the quality of tomato fruits due to its involvement in the regulation of many metabolic pathways that might be responsible for enhancing the nutritional characteristics as well as the defense of fruits.

## Abstract 2

Combining bioinformatics tools with metabolomics showed that foliar spray with chitosan increased the level of valuable compounds in tomato fruits.

## Graphical abstract

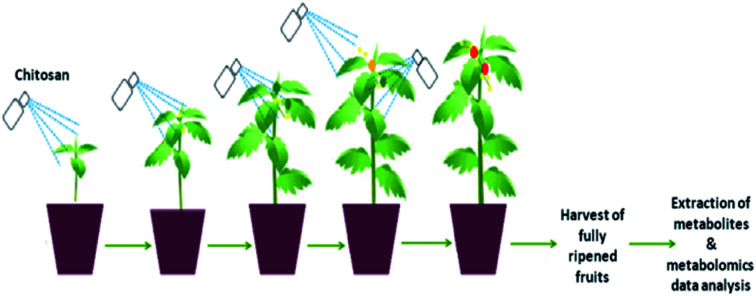

## 1. Introduction

Agrosystems can change over time due to the increase in drought, salinity, heat, metal toxicity, and mineral deficiency or by infection with pathogens.^1,2^ The use of synthetic molecules to overcome some of these issues is not always the best solution because of its feature to appear as a double edged-sword. As an example, pesticides from the 1940s are considered the best phytochemicals due to their ability to enhance either the quantity and the quality of important crops or the protection of plants against diseases, pests, fungus, and weeds.^3^ Despite their benefits, however, their excessive use, especially by exceeding the dose line to have more productivity, has developed into another issue as the deterioration of human health and the environment.

Recently, many researchers have focused on the valorization of shellfish waste to produce a natural fertilizer, such as chitosan, which therefore reduces the pollution generated by this waste, replaces pesticides, and increases plant growth and productivity.^4^

Chitosan, an aminopolysacharide, is a deacetylated form of chitin which is the second major polysaccharide in nature after cellulose.^5^ The application of chitosan in different fields has been raised by the employment of its diverse biological properties, such as non-toxicity and biodegradability. In agriculture, this polymer has been demonstrated to be a good elicitor due to its ability to stimulate the defense mechanisms and to protect the plant against pathogens.^6^ Among the mechanisms underlying its effect, there is a faster accumulation of Ca^2+^ in plant cells which then stimulates the stomata closure and therefore stops and limits the invasion of microorganisms inside the plant.^7^ It could also prime the plant by either the activation of defense hormones, such as jasmonic acid, or the induction of pathogenesis related-genes, such as chitinase and β-glucanase.^8^ Besides strengthening plant defense, the stimulation of defense metabolites as well as the limitation of the assimilation of CO_2_ because of stomata closure will adversely affect productivity. In contrast, it has been demonstrated that chitosan is a good biostimulator for plant growth. It could increase yield and improve the quality of fruits and vegetables of many species by enhancing the production of secondary metabolites, such as phenolic and terpenoid compounds, vitamin C, and lycopene.^8^ Therefore, it seems that chitosan prepares plants to be ready and adapt in any steady and urgent circumstance with a positive effect on plant growth and fruits quality.

The effect of foliar application of chitosan during seedling and fruit development on the production of secondary metabolites in fruits has not been yet studied. Therefore, a deep analysis of fruit metabolome is required to understand chitosan effects and to know, how to enhance either the esthetic or the taste quality of fruits.

Nowadays, many analytical techniques, such as HPLC/MS and NMR, were used to describe the chemical composition in varieties, to detect adulteration, and to discover new metabolites.^9^ Untargeted metabolomics, using liquid chromatography with mass spectrometry (LC MS/MS), is among the efficient analytical techniques, which may help to detect unforeseen changes in the food metabolome.^10^ This technique has been employed to compare volatile metabolites of 94 ripe tomato genotypes and to investigate the mechanisms and metabolic regulation that influence fruit quality.^11,12^ Therefore, we used LC-MS in combination with bioinformatics tools as an approach to perform non-targeted metabolite analyses of fruits from plants treated with different concentrations of chitosan. Resulting differences in the relative abundance of several metabolites showed the effects of chitosan application on tomato fruit metabolome.

## 2. Materials and methods

### 2.1. Chitosan preparation

Chitosan, with a 16% acetylation degree, has been produced from shrimp (*Parapenaeus longirostris*) shell waste, then, different doses (0, 0.25, 0.50, 0.75, and 1 mg ml^−1^) were prepared as described by El Amerany *et al.* (2020).^13^

### 2.2. Growing conditions and chitosan application

Tomato seeds (*Solanum lycopersicum* cv. *Campbell33*) were germinated in a commercial peat substrate at 28 °C. After 10 days, the seedlings were transplanted into plastic pots (4 kg) that contained a mixture of sand and peat (2 : 1) and then placed in a greenhouse with 24 °C temperature, 330 μmol m^−2^ s^−1^ photosynthetic photon flux density, and 69% relative humidity. At the four-leaf stage, uniform plants were chosen and divided into five groups which represent chitosan treatments at different concentrations (0, 0.25, 0.50, 0.75, and 1 mg ml^−1^) Then, plants were sprayed once every two weeks for three months either with chitosan or distilled water. Three replicates of each treatment were applied. Fully ripened fruits were harvested, cut into pieces, immediately frozen, and ground in liquid nitrogen followed by lyophilization.

### 2.3. Metabolite extraction

Extraction of metabolites was performed using 25 mg of freeze-dried fruit material. In cryotube (1.6 ml; Precellys Steel Kit 2.8 mm, Peqlab Biotechnologie GmbH, Erlangen, Germany), material was mixed in a bead mill (5.0 m s^−1^; 20 s; FastPrep24, MP Biomedicals LLC, Santa Ana, California) with 900 μl of a cold mixture of dichloromethane:ethanol (2 : 1, −80 °C) and 200 μl of trifuoroacetic acid (TFA, pH 1) for three times. The homogenized samples were centrifuged at 12 623 g for 3 min at 4 °C. The upper phase of each sample (≈200 μl, aqueous phase) was removed, while the rest was mixed again with 50 μl of TFA. After elimination of the aqueous phase of the second extraction, 600 μl of the lower phase (organic phase) was collected and transferred to a new tube. Then, 500 μl of tetrahydrofuran was added to the rest. After a fast mixture and centrifugation, the supernatant was collected and combined with the previous organic extract and dried in a nitrogen stream.

### 2.4. Analysis of untargeted metabolites using LC MS/MS

Dried extracts were resuspended in 180 μl 80% MeOH and centrifuged. Per sample 5 μl supernatant was injected for UPLC analysis. The separation of extracted metabolites was carried out using a Waters ACQUITY UHPLC system, equipped with a Nucleoshell RP18 column (2.1 × 150 mm, particle size 2.1 μm, Macherey and Nagel, GmbH, Düren, Germany), a binary solvent manager, and an ACQUITY sample manager (Waters GmbH, Eschborn, Germany). The mobile phase A consisted of 0.3 mmol l^−1^ of ammonium formate (NH_4_HCO_2_, pH 3.5 with formic acid), while the mobile phase B was acetonitrile. A gradient elution was as follow: 2 min, 5% B; 2 to 19 min, 95% B; 19 to 21 min, 95% B; 21.01 to 24 min, 5% B. The flow rate was 400 μl min^−1^ and the temperature was 40 °C. The metabolites were ionized by electrospray ionization in positive and negative mode.

**Fig. 1:**
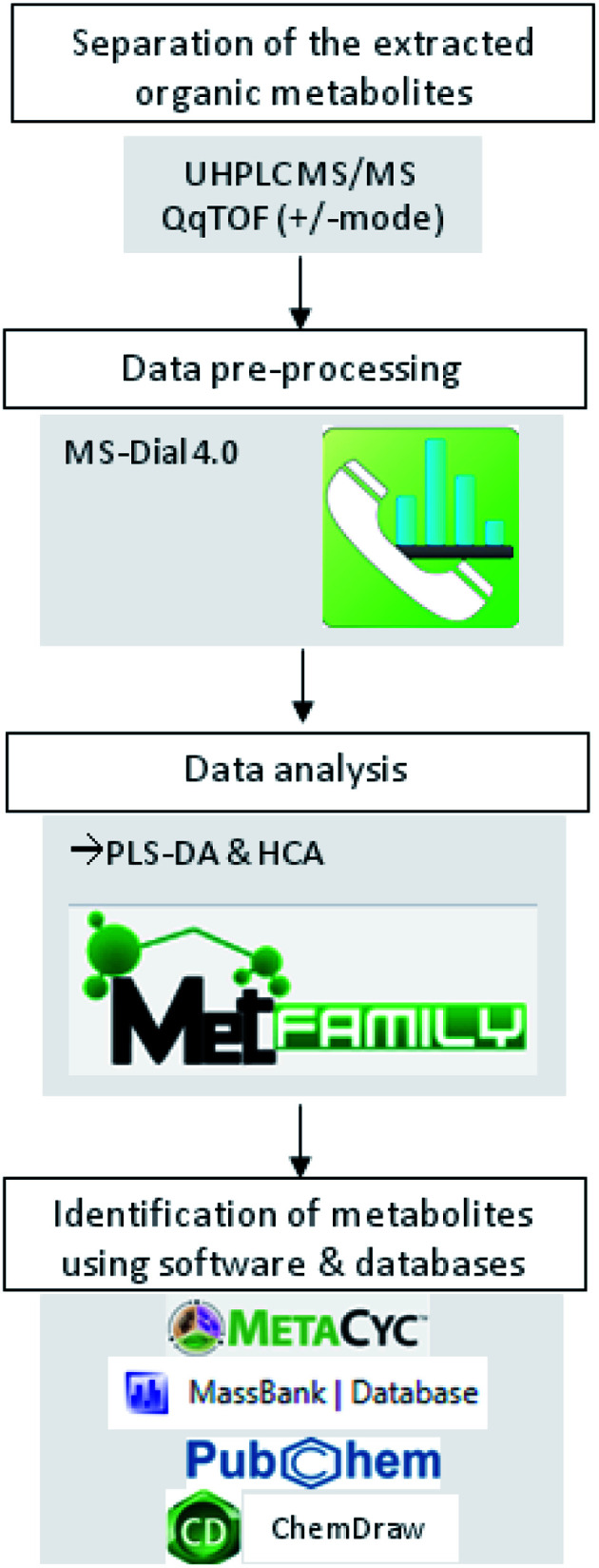
Workflow of metabolomics data analysis.

Mass spectrometric analysis of small molecules was performed by MS-TOF-SWATH-MS/MS (TripleToF 5600, both AB Sciex GmbH, Darmstadt, Germany) operating in negative or positive ion mode and controlled by Analyst 1.6 TF software (AB Sciex GmbH, Darmstadt, Germany) (Fig. 1). The source operation parameters were as the following: ion spray voltage, −4500 V/+5500 V; nebulizing gas, 60 psi; source temperature, 600 °C (600 TripleToF); drying gas, 70 psi; curtain gas, 35 psi. TripleToF instrument tuning and internal mass calibration were performed every 5 samples with the calibrant delivery system applying APCI negative or positive tuning solution, respectively (AB Sciex GmbH, Darmstadt, Germany).

TripleToF data acquisition was performed in MS1-ToF mode and MS2-SWATH mode. For MS1 measurements, ToF masses were scanned between 65 and 1250 Dalton with an accumulation time of 50 ms and a collision energy of 10 V (−10 V). MS2-SWATH-experiments were divided into 26 Dalton segments of 20 ms accumulation time each. Together the SWATH experiments covered the entire mass range from 65 to 1250 Dalton in 48 separate CID scan experiments, which allowed a cycle time of 1.1 s. For all MS/MS scans a declustering potential of 35 (or −35 V) was applied. Collision energies for all SWATH-MS/MS were set to 35 V (−35 V) and a collision energy spread of ±25 V, maximum sensitivity scanning, and elsewise default settings.

### 2.5. Annotation of metabolites

Prior metabolites identification and statistical analysis, raw data pre-processing were done using MS-Dial (version 4.0) (Fig. 1). Then, 2139 precursor ions detected in negative and positive ionization modes were filtered using two parameters: MS1 abundance threshold of 5000 counts and log2-fold change (LFC) of 0.5.

**Fig. 2:**
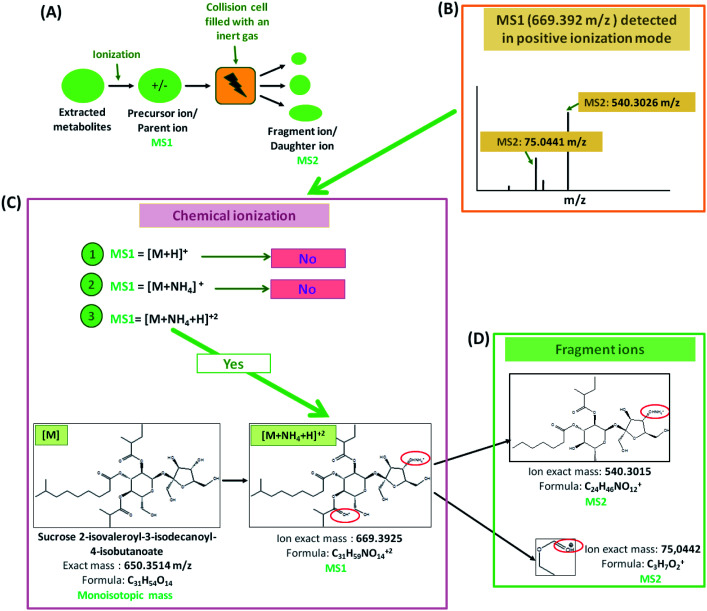
LC-MS based metabolomics procedure. (A) Annotation of untargeted metabolites is based on the identification of the precursor ions or parent ions (MS1) formed during the ionization of the extracted metabolites as well as their fragment ions or daughter ions (MS2) formed in the collision cell of TOF MS. (B) For instance, in positive ionization mode, a precursor ion which has an *m*/*z* value equal to 669.392 *m*/*z* is detected. (C) For this precursor ion, 3 chemical ionizations are possible, such as [M + H]^+^, [M + NH_4_]^+^, and [M + NH_4_+H]^2+^ and; therefore, the monoisotopic mass [M] of these 3 propositions can be calculated. Then, databases, especially Metacyc, Pubmed and MassBank, revealed that the calculated [M] of the proposition number 3 is the only one known as sucrose 2-isovaleroyl-3-isodecanoyl-4-isobutanoate. (D) Finally, using ChemDraw fragmentation tool, two daughter ions which are similar to those obtained by TOF MS are obtained and therefore, confirm that this molecule belongs to Acylsugars.

The identification and the annotation of metabolites were performed as described in Fig. 1 and 2. Briefly, to annotate metabolites, the monoisotopic mass of the precursor ions (MS1; [M + H]^+^, [M + NH_4_]^+^, [M − H]^−^ adducts) was filtered from the publicly available database (*i.e.*, Metacyc). If this mass matched a known metabolite, then a putative identification would be possible. Then, the identification of MS1 ions was confirmed if the mass of fragment ions detected by TOF MS was similar to those found either in reference spectra (MassBank and PubChem) or by ChemDraw software. Metabolite annotation was performed using a “MetFamily” web application, which is available free of charge at http://msbi.ipb-halle.de/MetFamily/. This website provides a dynamic link between MS1 data (*m*/*z*, retention time, and relative abundance) and its corresponding MS/MS spectra.

Moreover, in this study, 232 metabolites were identified (Tables S1 and S2†) and 16 of them were selected because of their importance in human diet as well as their high level in fruits from non treated plants or treated plants with chitosan, especially 0.75 mg ml^−1^.

### 2.6. Statistical analysis

Graphical representations, such as partial least squares regression in the discriminant analysis (PLS-DA) and hierarchical cluster analysis (HCA), were created to visualize the correlation between treatments and metabolites extracted. PLS-DA and HCA were performed using MetFamily web application (Fig. 1). Moreover, the analysis of a significant difference in metabolite abundance was done by CoStat 6.400 (CoHort software), using Duncan's test at *p* ≤0.05.

## 3. Results

### 3.1. Foliar spray with chitosan changes metabolite profile of tomato fruits

Tomato fruits from plants treated with chitosan treatments at different concentrations (0, 0.25, 0.50, 0.75, and 1 mg ml^−1^) were used for extraction of metabolites. The extracts were analyzed by electrospray ionization mass spectrometry (ESI-MS/MS) in negative and positive mode and revealed 713 and 1426 metabolite features per sample, respectively.

#### 3.1.1. Identification of metabolite families

To identify fruit metabolites with altered levels due to treatment with chitosan as well as metabolic pathways that were altered by the application of chitosan, the metabolomics data set for both ionization modes (positive and negative) were analyzed. Due to a large number of data, we used multidimensional statistical analysis to simplify the information obtained. In addition, graphic representations using Partial Least Squares Discriminant Analysis (PLS-DA) and Hierarchical Cluster Analysis (HCA), were generated to show similarities and dissimilarities between fruit groups (Ch0, Ch0.25, Ch0.50, Ch0.75, and Ch1).

**Fig. 3:**
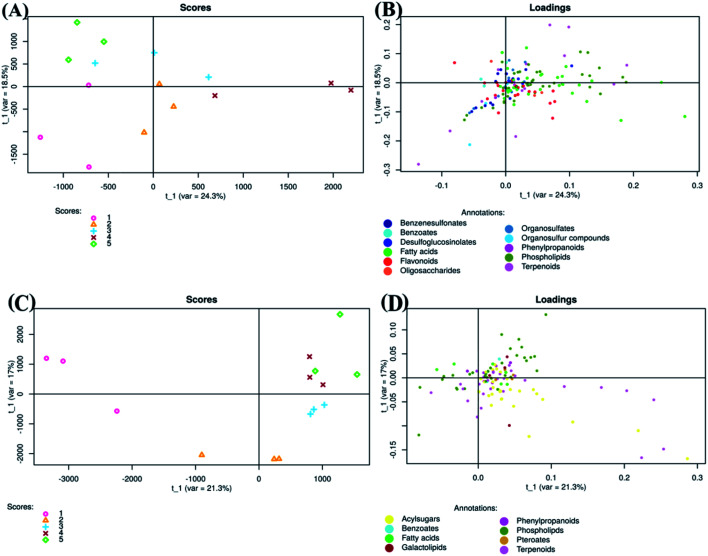
Partial least squares discriminant analysis of metabolites detected in negative (A and B) and positive (C and D) ion mode LC MS/MS. Scores (1 : 0 mg ml^−1^; 2 : 0.25 mg ml^−1^; 3 : 0.50 mg ml^−1^; 4 : 0.75 mg ml^−1^; 5 : 1 mg ml^−1^).

Regarding negative ion mode data, PLS-DA score plot showed that samples of each group were clustered together and separated from others (Fig. 3A). The total variation between those groups was equal to 42.8% (PLS factor 1 (*t*_1_): 24.3% + PLS factor 2 (*t*_2_): 18.5%). Additionally, fruits treated with Ch0.75 (group 4) were so strongly different from those of control (group 1). However, fruits treated with other concentrations of chitosan were clustered closely to non-treated fruits.

To clarify the reason for discrimination between samples, the loadings plot was created (Fig. 3B). This plot revealed that the metabolites were frequently localized on the right side which therefore confirms the positive correlation between these metabolites and samples treated with Ch0.75.

Concerning positive ion mode data, the score plot (Fig. 3C) showed that groups 4 and 5 (1 mg ml^−1^ chitosan) were closed to each other on both axis (PLS1 (21.3%) and PLS2 (17%)), which therefore indicates a high correlation between them. However, samples of non-treated fruits (group 1) were clustered far from those treated with chitosan (group 2, 3, 4, and 5), indicating a strong negative correlation between treated and non-treated fruits. Loadings plot showed also that metabolites detected in positive ion mode are less scattered, in comparison to those detected in negative ion mode (Fig. 3B and D).

**Fig. 4:**
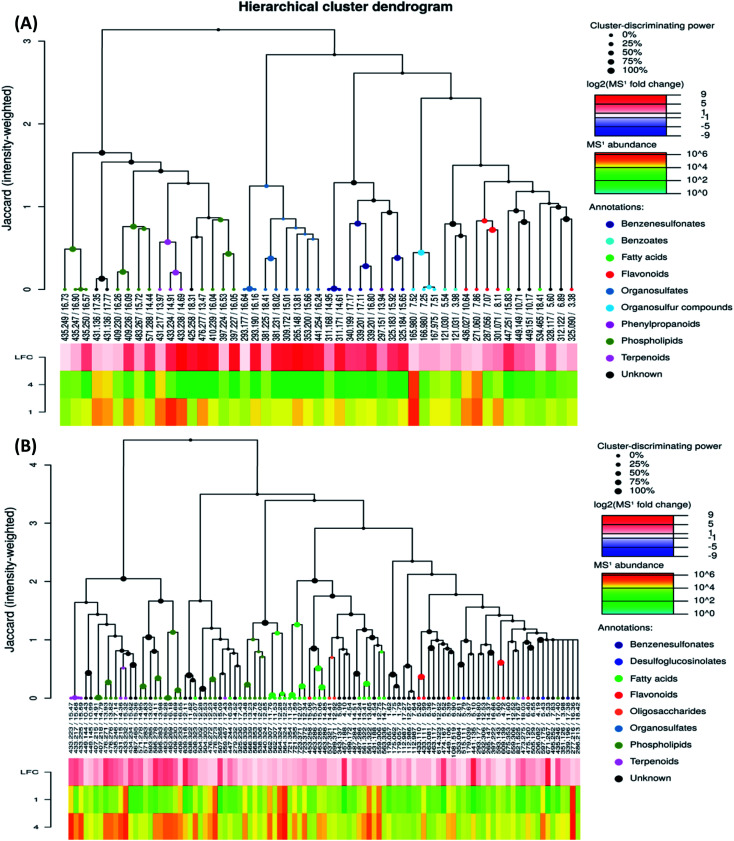
Hierarchical cluster analysis of precursor ions detected in negative mode. To evaluate chitosan effects on metabolites detected, 713 ions were filtered using two parameters: MS1 abundance threshold of 5000 counts and log2-fold change (LFC) of 0.5. (A) Represents clusters that were abundant in group 1 (fruits from non-treated plants) in comparison to group 4 (fruits from treated plants with 0.75 mg ml^−1^ of chitosan), while (B) represents the clusters that are abundant in group 4 in comparison to group 1.

**Fig. 5:**
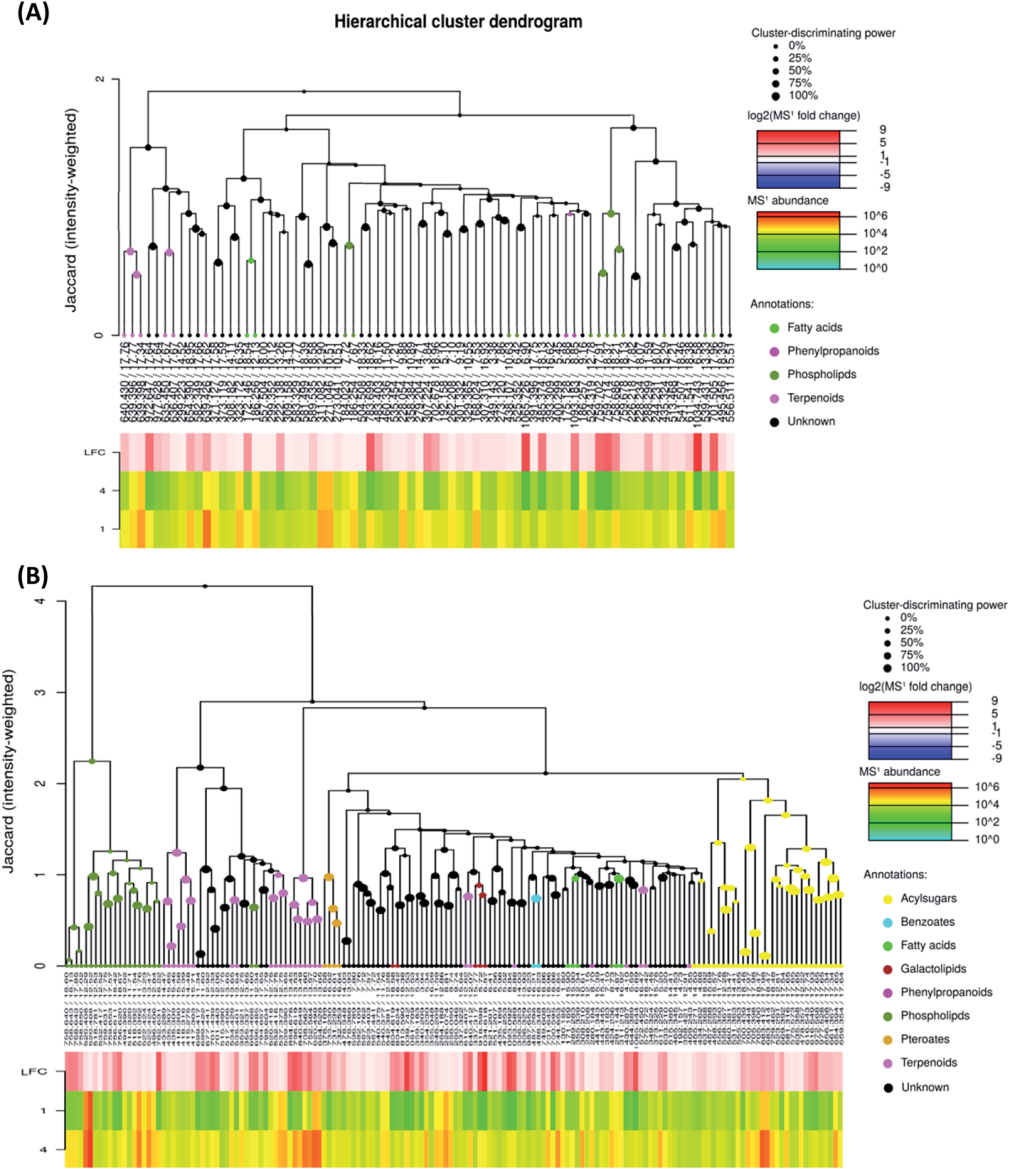
Hierarchical cluster analysis of precursor ions detected in positive mode. To evaluate chitosan effects on metabolites detected, 1426 ions were filtered using two parameters: MS1 abundance threshold of 5000 counts and log 2-fold change (LFC) of 0.5. (A) Represents clusters that were abundant in group 1 (fruits from non-treated plants) in comparison to group 4 (fruits from treated plants with 0.75 mg ml^−1^ of chitosan), while (B) represents the clusters that are abundant in group 4 in comparison to group 1.

To investigate the difference in the metabolome of treated (0.75 mg ml^−1^ chitosan) and non-treated fruits, HCA plots were created (Fig. 4 and 5). A first filer setting (average MS1 abundance is equal to 5000) was performed to reduce the number of ions analyzed to 232 and 560, in negative and positive ion mode, respectively. The annotation of negative ions using HCA spectra, showed that samples were composed of several metabolites belonging to eleven main classes (*i.e.*, benzenesulfonates, benzoates, desulfoglucosinolates, fatty acids, flavonoids, oligosaccharides, organosulfates, organosulfur compounds, phenylpropanoids, phospholipids, and terpenoids) (data not shown). Then, a second filter setting (fold change is equal to 1.41) was used to define metabolites that their abundances were decreased (Fig. 4A and 5A) or increased (Fig. 4B and 5B) in fruits treated with Ch0.75 in comparison to non-treated fruits. Concerning non-treated fruits, the number of metabolites was decreased to achieve 49 and two metabolite families (desulfoglucosinolates and oligosaccharides) disappeared (Fig. 4A). However, Fig. 4B revealed the abundance of 103 metabolites that were higher in treated fruits with the reduction of levels of three metabolite families (*i.e.*, benzoates, organosulfur compounds, and phenylpropanoids).

Moreover, a few parent classes (8 classes) were identified in positive ion mode (Fig. 5A and B). Five of these classes were most common in both ionization modes; however, the remaining three classes (*i.e.*, acylsugars, galactolipids, and pteroates) were new and abundant in group 4 than group 1.

#### 3.1.2. Identification of feature

**Fig. 6:**
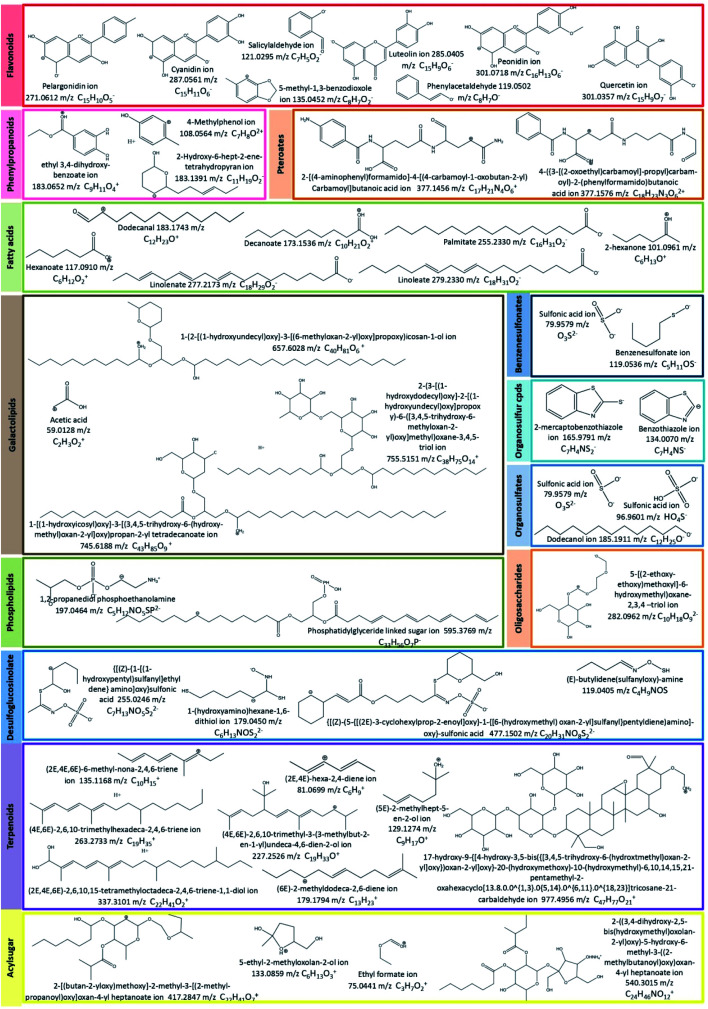
Metabolites markers used for metabolomics data analysis.

The annotation of ions presented in Tables S1 and S2† was done based on the similarity between the monoisotopic mass of precursor ion detected by mass spectrometry and those found in the database (theoretical mass) as well as their fragment ions. Fig. 6 lists some product ions used for metabolomics data analysis.

For instance, some flavonoids that have a positive charge were detected in negative ion mode, as anthocyanidins linked sugar (peak 22 and 23), pelargonidin (or luteolindin) (peak 113), cyanidin (peak 114), and peonidin (peak 115). The masse fragment ion found at *m*/*z* 271.0600 (C_15_H_10_O_5_^−^) for peaks 23 and 113 was attributed to pelargonidin ion. Moreover, fragment ions found at *m*/*z* 287.0572 (C_15_H_11_O_5_^−^) and *m*/*z* 301.0768 (C_16_H_13_O_6_^−^) were attributed to cyanidin and peonidin ions, respectively. These ions were generated after the deprotonation of a hydroxyl group which then switch one carbon double bond to a single bond (Fig. 6).

Additionally, different molecules that have a negative change were identified, as rutin (peak 25). The annotation of this peak was done based on the similarity between the monoisotopic mass of precursor ion detected (*m*/*z* 610.1572) and of theoretical mass (*m*/*z* 610.1534; C_27_H_30_O_16_) as well as the presence of deprotonated fragment ion of quercetin (*m*/*z* 301.0327; C_15_H_9_O_7_^−^) (Fig. 6).

### 3.2. Action mode of chitosan

**Fig. 7:**
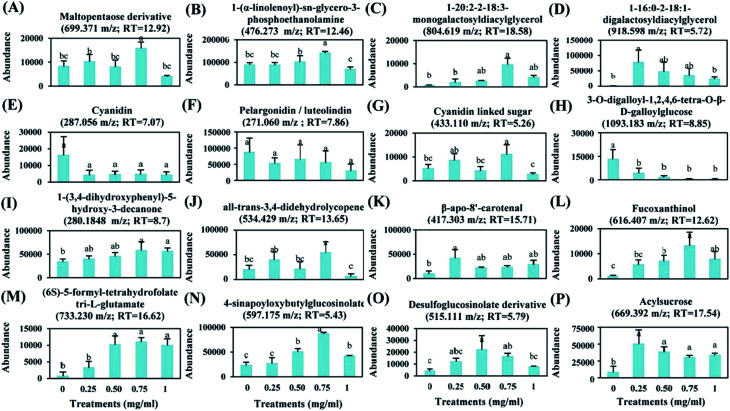
Changes in abundance of 16 metabolites (A–P) detected in tomato fruits under chitosan application. Data represents average of 3 replicates (±SD). Treatments having different lowercase letters are significantly different using one-way ANOVA and Duncan test (*p* ≤0.05).

To evaluate the effect of foliar application of chitosan on tomato fruit quality, sixteen metabolites were selected based on their major abundance either in fruits from treated or non-treated plants (Fig. 7).

Application of 0.75 mg ml^−1^ chitosan had a positive effect on the levels of sugars, fatty acids, and lipids. Here, fruits from treated plants showed a significant increase in the relative abundance of maltopentaose derivative (9%), 1-(α-linolenoyl)-*sn*-glycero-3-phosphoethanolamine (58%), monogalactosyldiacylglycerol (MGDG) (1446%), and digalactosyldiacylglycerol (DGDG) (8231%) in comparison to fruits from untreated plants (Fig. 7A–D). Additionally, the levels of anthocyanidins (cyanidin and pelargonidin) were not changed by chitosan application (Fig. 7E and F); however, the abundance of cyanidin linked sugar was induced by about 116% in fruits from treated plants compared to fruits from non-treated plants (Fig. 7G).

The relative abundance of phenylpropanoid compounds, such as 3-*O*-digalloyl-1,2,4,6-tetra-*O*-β-d-galloylglucose, was reduced by 67 to 95% in fruits from treated plants, compared to fruits from non-treated plants (Fig. 7H). However, the level of 1-(3,4-dihydroxyphenyl)-5-hydroxy-3-decanone was induced by about 69% in fruits from plants treated with 0.75 mg ml^−1^ of chitosan (Fig. 7I).

Moreover, a significant increase in the relative abundance of terpenoids, such as all-*trans*-3,4-didehydrolycopene (170%), β-apo-8′-carotenal (132%), and fucoxanthinol (1075%) was observed due to plant's treatment with 0.75 mg ml^−1^ of chitosan (Fig. 7J–L). Also, this treatment stimulated the accumulation of (6*S*)-5-formyl-tetrahydrofolate tri-l-glutamate, 4-sinapoyloxybutylglucosinolate, and desulfoglucosinolate derivative which their levels were increased by about 1444%, 274%, and 293% in fruits from treated plants, respectively, compared to the control (Fig. 7M–O). Furthermore, our data showed that acylsugar levels were increased by about 506%, 362%, 263%, and 304% in fruits from plants treated with 0.25, 0.50, 0.75, and 1 mg ml^−1^ of chitosan, respectively, in comparison to fruits from non-treated plants (Fig. 7P).

## 4. Discussion

In this study, we addressed the question, whether foliar spray with chitosan affects the quality of tomato fruits. Performing a non-targeted metabolite profiling approach on mature tomato fruits from plants treated or not with different concentrations of chitosan led to the detection of many signals by LC-MS. Bioinformatics analyses revealed differences in the metabolome of fruits from chitosan-treated plants in comparison to those from non-treated plants being related to some specific metabolites, which might change the aesthetic of fruits (color and flavor), increase their defense, and improve their nutritional values.

### 4.1. Chitosan impacts on colors and flavors of tomato

Anthocyanins are classified as important flavonoids, derived from phenylpropanoid pathway. The majority of anthocyanins produced by plants provide the dark color of purple, blue, and red.^14^ In our study, chitosan application to tomato fruits increased the level of cyanidin-3-*O*-β-d-glucoside (CG) and pelargonidin-3-*O*-β-d-glucoside (PG) and decreased the level of phlorizin, pelargonidin, cyanidin, and peonidin (Tables S1 and S2†). The increase in the content of anthocyanidins decorated with sugars could be due to the activation of UDP-glucose:flavonoid-3-*O*-glycosyltransferase enzyme (UFGT).^14,15^ It is known that accumulation of anthocyanins can be increased in response to abiotic stress, but also treatment of strawberry fruits with chitosan led to enhanced levels of two anthocyanins.^16^ Usually, anthocyanins are less available in the skin of some fruits and vegetables (*i.e.*, grape, eggplant, blueberry, potato, purple potato, and red cherry), and not present in other edible products that are consumed widely, as tomato.^17^ Due to their great role to overcome a range of chronic diseases, several attempts have been done to raise anthocyanins levels in fruits. Despite the applying of transgenic approaches to improve the anthocyanin composition in tomatoes,^18^ the biochemical and genetic complexity of the fruits made gene selection for improving anthocyanins levels extremely difficult. Therefore, treatment of plants with chitosan might provide a simple way to improve anthocyanins levels in tomato.

**Fig. 8:**
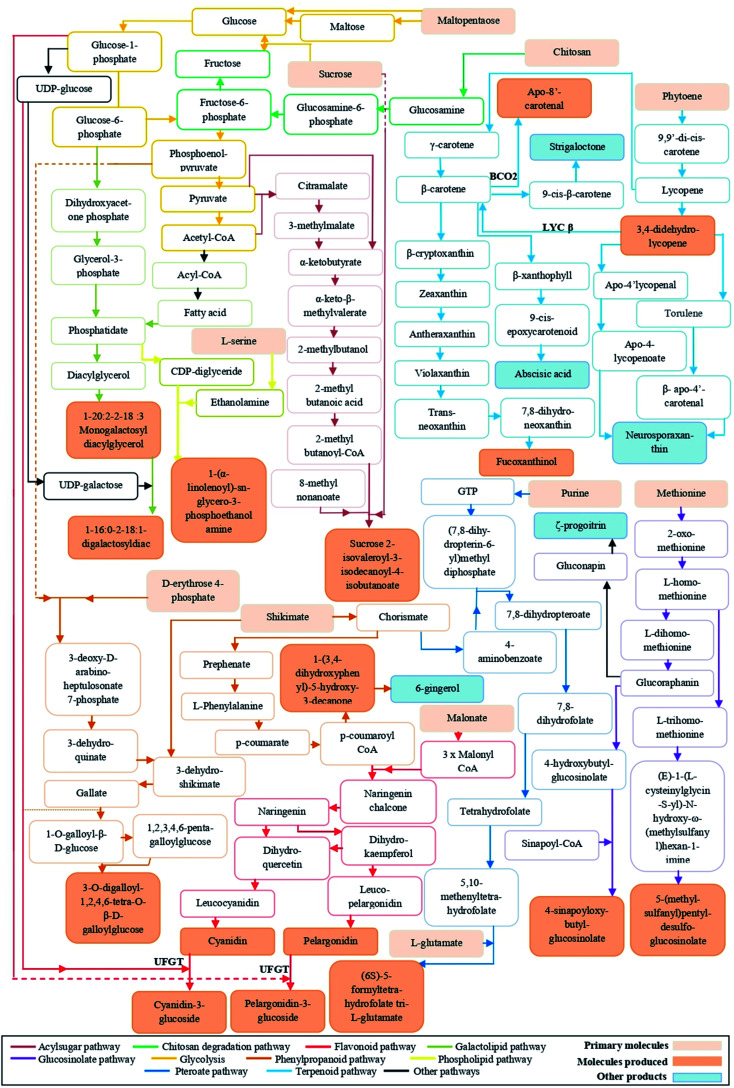
A Putative model summarizing the metabolic pathways affected by chitosan. This model is based on well-known metabolic pathways, such as KEGG and Metacyc. Orange boxes represent metabolites stimulated by the application of chitosan. Pink boxes represent the precursors implicated in the generation of these metabolites. Blue boxes represent other products that are not affected by the application of chitosan.

In addition, metabolomics data analysis revealed that chitosan application to tomato fruits increased the level of *trans*-3,4-didehydrolycopene and β-apo-8′-carotenal. These two compounds not only give orange pigments, but also can reduce the risk of cancer and heart diseases.^19^ Recent studies reported that chitosan elicited lycopene and carotenoid biosynthesis.^8,20^ To our knowledge, the accumulation of carotenoid derivatives (*trans*-3,4-didehydrolycopene and β-apo-8′-carotenal) appears for the first time in tomato. Although chitosan mechanisms, involved in the biosynthesis of these metabolites, are unclear, we made a hypothetical biochemical pathway in Fig. 8.

Besides the colors, flavors are among the characteristics that have an impact on the quality of fruits. To date, over 400 flavors have been discovered in mature tomato fruits,^21^ but with relatively low levels.^22^ The reasons of this minority can be attributed to either a genetic aspect or biochemical changes that taking place during post-harvest and handling practices.^22^ However, metabolomics analysis of our data showed the presence of a molecule that is very close to 1-(3,4-dihydroxyphenyl)-5-hydroxy-3-decanone, a particular precursor of ginger flavor (Fig. 8). Chitosan effect on tomato fruits flavor has not yet been documented. But, the enhancement of the level of this flavor could be explained by the fact that chitosan application to fruits could make a barrier allowing to keep this aroma longer.^23^

### 4.2. Chitosan induces the biosynthesis of defense metabolites

Chitosan is known to be an elicitor that stimulates the natural defense mechanisms of plants.^6^ The analysis of metabolomics profiles showed that the relative abundance of sucrose 2-isovaleroyl-3-isodecanoyl-4-isobutanoate, a class of acylsugar, was increased in all fruits of chitosan-treated plants. Acylsugars are sticky molecules synthesized and stored in the glandular trichomes type IV of tomato leaves and fruits to enhance plant resistance against herbivores and micro-organisms infection.^24^ In *Artemisia annua* L., Kjær *et al.* (2012)^25^ showed that foliar application of chitosan increased the leaf trichome density. Therefore, we suppose that its application to leaves could stimulate the biosynthesis of acylsucrose even in fruits. Besides acylsugar, other defense compounds identified were gallotanins. The defense mechanisms of these compounds are related to their ability to form complexes with proteins and lipids.^26^ However, as it shows in Fig. 7, chitosan application had a negative impact on the level of 3-*O*-digalloyl-1,2,4,6-tetra-*O*-β-d-galloylglucose. The deactivation of gallotanins biosynthesis might be due to the involvement of sugars and fatty acids in biosynthesis pathways of flavonoids, acylsugars, and lipids (Fig. 8).

### 4.3. Improvement of the nutritional quality of tomato under chitosan application through the increase in the level of bioactive molecules deficient in fruits

Our study revealed that chitosan application increased the level of sulfur-containing glucosides: glucosinolates (GLs) (*i.e.*, 4-sinapoyloxybutylglucosinolate). Gls are one of the most abundant groups of phytochemicals in cruciferous plants, as broccoli (*Brassica oleracea* var. *italica*), cabbage (*B. oleracea* var. *capitata f. alba*), cauliflower (*B. oleracea* var. *botrytis*), rapeseed (*Brassica napus*), mustard (*Brassica nigra*), and horse-radish (*Armoracia rusticana*); however, rare groups of metabolites in tomato.^27^ Gls can break down into different products (*i.e.*, isothiocyanates, thiocyanates, oxazolidinethione, and epithionitriles) that are responsible for plant taste and odor.^28^ Meanwhile, other study has shown that the conversion of GLs to isothiocyanates in the small intestine and colon may help the body to detoxify the dangerous product and to treat cancer.^29^ Changes in environmental conditions (*i.e.*, temperature, heavy metal stress, and herbivore infections) were found to increase the contents of Gls.^30^ Our results were, however, incongruent with the fact that the application of 100 μg of elicitor (chitin or chitosan) to brassica seedlings did not change the content of glucosinolates.^31^

Additionally, folates are essential vitamins for human health due to their involvement in many biochemical processes, such as the carbon metabolism and the DNA biosynthesis.^32^ Although tomatoes are rich in bioactive molecules, they contain small amounts of folate. The increase in the consumption of food deficient in this molecule may cause several human disorders as cancer.^33^ Our results showed that there is an increase in the relative abundance of (6*S*)-5-formyl-tetrahydrofolate tri-l-glutamate in fruits from tomato plants treated with medium (0.50 mg ml^−1^) and high (0.75 mg ml^−1^ and 1 mg ml^−1^) doses of chitosan. Such an accumulation of folate under chitosan application could be attributed to the stimulation of the biosynthesis of chorismate, purine, and glutamate^34^ (Fig. 8). Therefore, the increase in native folate in tomato fruits could be considered as an alternative approach to avoid the fortification of fruits with synthetic folate (*i.e.*, pteroylmonoglutamyl folate).^35^

### 4.4. Chitosan could delay the deterioration of fruits

The metabolomics data analysis revealed the presence of two classes of galactolipids (monogalactosyldiglyceride (MGDG) and digalactosyldiglyceride (DGDG)). These compounds are the major lipid component in photosynthetic tissues (chloroplasts) of higher plants.^36^ Moreover, Whitaker (1986) showed that the mature-green tomato contained almost 70% of galactolipids, in comparison to leaf chloroplasts.^37^ Other studies have reported that ripening of fruit did not alter the content of simple compounds (*i.e.*, fatty acids and triacylglycerols); in contrast, it affected the level of large molecules (*i.e.*, phospholipids and galactolipids) that were dropped.^38,39^ Thus, our results might suggest that chitosan application could delay the fruit softening process which is usually caused by the degradation of phospholipids and triacylglcerols, and therefore could prevent fruit spoilage.^40^

## 5. Conclusions

The analysis of metabolomics data is a successful strategy that helped us to specify which features of tomato fruits were changed by the application of chitosan to leaves. The aesthetic, the nutritional, and the defense properties of tomatoes were promoted by foliar application of chitosan to tomato plants. With these data, our understanding of the effects of this polymer on tomato plants has been increased. However, additional analyses as the quantification of metabolites related to those properties will be useful to prove these findings. On the other hand, analyzing metabolomics data enabled us to identify some specific ion markers that we can used as libraries to examine food ingredients.

## Author contributions

F. E. A carried out the experiment, worked in data curation, and analyzed data, and wrote the original draft. All authors contributed to the conception and design of the study, the analysis and discussion of the results, and the revision of the manuscript. All authors have read and agreed to the published version of the manuscript.

## Conflicts of interest

There are no conflicts to declare.

## Supplementary Material

RA-011-D1RA05865G-s001
