# Supplementary material for: Use of metabolomics data analysis to identify fruit quality markers enhanced by the application of an aminopolysaccharide
Source: RSC Adv. 2021 Nov 3;11(56):35514–24. doi: 10.1039/d1ra05865g (PMC9043226; doi:10.1039/d1ra05865g)
Supplement: RA-011-D1RA05865G-s001 [file RA-011-D1RA05865G-s001.pdf]

Electronic Supplementary Material (ESI) for RSC Advances.
This journal is © The Royal Society of Chemistry 2021

Table S1: Identification of the set of metabolites found in fruits treated with 0.75 mg/ml of chitosan. Treatments having different lowercase letters are significantly different using one-way ANOVA and Duncan test (p ≤ 0.05)

	Compounds	Features	MS1 Feature	RT (min)	Ion mode	Monoisotopic mass			Fragments (MS/MS)			Smiles	InChI	Abundance in fruits from treated plants with chitosan ($mg.mL^{-1}$)				
						Detected	Database	Formulae	Detected	Database	Formulae			0	0.25	0.50	0.75	1
Benzenesulfonates	4-(3-butylnonyl)-benzensulfonic acid	1	339.196	17.38	Negative	***	340.2072	$C_{19}H_{32}O_3S$	325.1779<br>220.1476<br>183.0132<br>119.0502<br>79.9586	325.1843<br>220.1308<br>183.0132<br>119.0536<br>79.9579	$C_{18}H_{29}O_3S^-$<br>$C_{14}H_{20}S^{4-}$<br>$C_8H_7O_3S^{3-}$<br>$C_5H_{11}OS^-$<br>O_3S^{2-}	OS(C1=CC=C(CCC(CCCCCC)CCCC)C=C1)(=O)=O	-	7135b	22006a	30186a	18196ab	17646ab
Benzoates	Dihydroxyphenyl-5-methyloxazoline-norspermidine-2,3-dihydroxybenzoate	2	487.298	16.21	Positive	***	486.2114	$C_{24}H_{30}N_4O_7$	486.3007<br>351.2350<br>147.1190<br>133.1300<br>105.1019<br>91.0873	486.3043<br>351.2391<br>147.1128<br>133.1335<br>105.1022<br>91.0866	$C_{23}H_{42}N_4O_7^{2+}$<br>$C_{18}H_{31}N_4O_3^+$<br>$C_6H_{15}N_2O_2^+$<br>$C_6H_{17}N_2O^+$<br>$C_4H_{13}N_2O^+$<br>$C_3H_{11}N_2O^+$	C[C@H]2(OC(/C1(\C=C/C(/O)=C(/C=1)/O))=N/C2C(=O)NCCCNCCCNC(=O)C3(/C=C\C=C(C(\O)=3)\O))	InChI=1S/C24H30N4O7/c1-14-20(28-24(35-14)15-7-8-17(29)19(31)13-15)23(34)27-12-4-10-25-9-3-11-26-22(33)16-5-2-6-18(30)21(16)32/h2,5-8,13-14,20,25,29-32H,3-4,9-12H2,1H3,(H,26,33)(H,27,34)/t14-,20?/m1/s1	1839b	2506b	3038b	5597ab	20193a
		3	488.348	16.23	Positive	***	486.2114	$C_{24}H_{30}N_4O_7$						4796b	8609ab	11926a	9638ab	7875ab
Desulfoglucosinolates	4-sinapoyloxybutylglucosinolate	4	597.175	5.43	Negative	***	597.1186	$C_{22}H_{31}NO_{14}S_2$	477.1587<br>255.0308<br>119.0383	477.1502<br>255.0246<br>119.0405	$C_{20}H_{31}NO_8S_2^{2-}$<br>$C_7H_{13}NO_5S_2^{2-}$<br>C_4H_9NOS	COC2(/C=C(/C=C/C(OCCCCC(/S[C@@H]1(O[C@H](CO)[C@@H](O)[C@H](O)[C@@H](O)1))=N\OS(=O)(=O)[O-])=O)/C=C(C(\O)=2)/OC)	InChI=1S/C22H31NO14S2/c1-33-13-9-12(10-14(34-2)18(13)26)6-7-17(25)35-8-4-3-5-16(23-37-39(30,31)32)38-22-21(29)20(28)19(27)15(11-24)36-22/h6-7,9-10,15,19-22,24,26-29H,3-5,8,11H2,1-2H3,(H,30,31,32)/p-1/b7-6+,23-16+/t15-,19-,20+,21-,22+/m1/s1	23263c	26701c	50648b	87047a	42042b
	5-(methylsulfanyl)pentyl-desulfoglucosinolate (I & II) derivative	5	515.111	5.79	Negative	***	***	***	293.1068<br>179.0370<br>173.0463	293.1130<br>179.0450<br>173.0521	$C_{12}H_{23}NO_3S_2^{2-}$<br>$C_6H_{13}NOS_2^{2-}$<br>$C_7H_{11}NO_2S^{2-}$	CSCCCCCC(/S[C@@H]1(O[C@H](CO)[C@@H](O)[C@H](O)[C@@H](O)1))=N\O	InChI=1S/C13H25NO6S2/c1-21-6-4-2-3-5-9(14-19)22-13-12(18)11(17)10(16)8(7-15)20-13/h8,10-13,15-19H,2-7H2,1H3/b14-9+/t8-,10-,11+,12-,13+/m1/s1	4169c	12016abc	22147a	16387ab	7743bc

**Phenylpropanoids**	1-(3,4-dihydroxy-phenyl)-5-hydroxy-3-decanone	6	280.184	8.7	Positive	***	280.1675	$C_{16}H_{24}O_4$	172.1449<br>116.0783<br>108.0507	172.1452<br>116.0826<br>108.0564	$C_{10}H_{20}O_2^{2+}$<br>$C_6H_{12}O_2^{2+}$<br>$C_7H_8O^{2+}$	CCCCC[C@H](O)C<br>C(=O)CCC1(\C=C(<br>C(/O)=C\C=1)\O)	InChI=1S/C16H24O4/c1-<br>2-3-4-5-13(17)11-<br>14(18)8-6-12-7-9-<br>15(19)16(20)10-12/h7,9-<br>10,13,17,19-20H,2-<br>6,8,11H2,1H3/t13-/m0/s1	34025b	39727ab	45349ab	57783a	56184a
**Fatty acids**	Linolenate conjugated	7	325.236	17.43	Negative	***	***	***	279.2312	279.2330	$C_{18}H_{31}O_2^-$	CC\C=C/C\C=C/C\C<br>=C/CCCCCCCC(=O<br>)[a glycerolipid]	-	5196ab	5622ab	4588b	8509a	5847ab
		8	447.244	16.39	Negative	***	***	***	279.2312	279.2330	$C_{18}H_{31}O_2^-$			3068b	9001ab	12803a	15119a	6521ab
		9	463.285	15.06	Negative	***	***	***	279.2312<br>255.2312	279.2330<br>255.233	$C_{18}H_{31}O_2^-$<br>$C_{16}H_{31}O_2^-$			13514c	21482bc	28089b	54438a	20630bc
		10	487.286	14.24	Negative	***	***	***	279.2312	279.2330	$C_{18}H_{31}O_2^-$			7810b	16461b	11777b	30702a	11314b
		11	559.306	12.75	Negative	***	***	***	277.2151	277.2173	$C_{18}H_{29}O_2^-$			57614b	49322b	169901a	132406a	54561b
		12	561.322	13.41	Negative	***	***	***	279.2312	279.2330	$C_{18}H_{31}O_2^-$			12810bc	33029ab	32821ab	44741a	8618c
		13	562.307	11.76	Negative	***	***	***	279.2312	279.2330	$C_{18}H_{31}O_2^-$			23339a	18663a	66075a	71246a	72596a
		14	564.324	12.28	Negative	***	***	***	277.2151	277.2173	$C_{18}H_{29}O_2^-$			75115b	71852b	50183b	127415a	63186b
		15	563.336	14.77	Negative	***	***	***	277.2151	277.2173	$C_{18}H_{29}O_2^-$			6570ab	12189a	7841ab	10895a	3274b
		16	721.354	11.34	Negative	***	***	***	277.2151	277.2173	$C_{18}H_{29}O_2^-$			7180b	4953b	14125a	12677a	6159b
		17	723.371	12.07	Negative	***	***	***	279.2312	279.2330	$C_{16}H_{31}O_2^-$			3994b	11723a	13222a	15189a	3043b
	12-oxo-9-hydroxy-10[E],15(Z)-octadecadi-enoate	18	311.213	16.92	Positive	***	310.2144	$C_{18}H_{30}O_4$	203.2080<br>177.1528<br>105.0926<br>77.0551	203.2006<br>177.1485<br>105.0910<br>77.0597	$C_{12}H_{27}O_2^+$<br>$C_9H_{21}O_3^+$<br>$C_5H_{13}O_2^+$<br>$C_3H_9O_2^+$	CC\C=C/CCC(=O)/<br>C=C/C(O)CCCCCC<br>CC(=O)[O-]	InChI=1S/C18H30O4/c1-<br>2-3-4-8-11-16(19)14-15-<br>17(20)12-9-6-5-7-10-13-<br>18(21)22/h3-4,14-<br>15,17,20H,2,5-<br>13H2,1H3,(H,21,22)/p-<br>1/b4-3-,15-14+	21169a	20147a	22425a	32376a	28937a
	2-oxo-behenate	19	355.339	12.31	Positive	***	354.3134	$C_{22}H_{42}O_3$	183.1714<br>172.1724	183.1743<br>172.1816	$C_{12}H_{23}O^+$<br>$C_{11}H_{24}O^{2+}$	C(CCCCCCCCCCC<br>CCCCCCCCCC([O-<br>])=O)=O	InChI=1S/C22H42O3/c23<br>-21-19-17-15-13-11-9-7-<br>5-3-1-2-4-6-8-10-12-14-<br>16-18-20-<br>22(24)25/h21H,1-<br>20H2,(H,24,25)/p-1	3620ab	5299ab	4757ab	7179a	1860b
	2-undecanone	20	171.169	15.90	Positive	***	170.1671	$C_{11}H_{22}O^+$	171.1665<br>101.0889<br>73.0470	171.1743<br>101.0961<br>73.0648	$C_{11}H_{23}O^+$<br>$C_6H_{13}O^+$<br>$C_4H_9O^+$	CCCCCCCCCC(=O<br>)C	InChI=1S/C11H22O/c1-<br>3-4-5-6-7-8-9-10-<br>11(2)12/h3-10H2,1-2H3	2533a	2978a	3066a	2711a	2596a
	Derivative	21	289.283	15.90	Positive	***	***	***	172.1724<br>171.1665	172.1816<br>171.1743	$C_{11}H_{24}O^{2+}$<br>$C_{11}H_{23}O^+$	-	-	2653b	2901b	119097a	2924b	2592b
**Flavonoids**	Anthocyanins<br>Cyanidin-3-O-β-D-glucoside	22	449.106	5.39	Negative	***	449.1078	$C_{21}H_{21}O_{11}^+$	449.1079<br>269.1109<br>135.0451<br>101.0393	449.1087<br>269.1183<br>135.0452<br>101.0608	$C_{21}H_{21}O_{11}^-$<br>$C_{17}H_{17}O_3^-$<br>$C_8H_7O_2^-$<br>$C_5H_9O_2^-$	C(O)[C@@H]1([C<br>@@H](O)[C@H](O<br>)[C@@H](O)[C@@<br>H](O1)OC3(\C(C2(\<br>C=C(C(/O)=C\C=2)\<br>O))=[O+]C4(\C(/C=<br>3)=C(/C=C(/C=4)/[<br>O-])\[O-])))	InChI=1S/C21H20O11/c2<br>2-7-16-<br>17(27)18(28)19(29)21(32<br>-16)31-15-6-10-12(25)4-<br>9(23)5-14(10)30-20(15)8-<br>1-2-11(24)13(26)3-8/h1-<br>6,16-19,21-22,27-<br>29H,7H2,(H3-<br>,23,24,25,26)/p-1/t16-,17-<br>,18+,19-,21-/m1/s1	18926a	29479a	11112a	34816a	7055a
	Cyanidin-3-O-β-D-											C(O)[C@@H]1([C<br>@@H](O)[C@H](O<br>)[C@@H](O)[C@@	InChI=1S/C21H20O11/c2<br>2-7-16-<br>17(27)18(28)19(29)21(32					

	glucopyrano-side											H](O1)OC3(\C(C2(\C=C(C(/O)=C\C=2)\O))=[O+]C4(\C(/C=3)=C(/C=C(/C=4)/[O-])\[O-])))	-16)31-15-6-10-12(25)4-9(23)5-14(10)30-20(15)8-1-2-11(24)13(26)3-8/h1-6,16-19,21-22,27-29H,7H2,(H3-,23,24,25,26)/p-1/t16-,17-,18+,19-,21-/m1/s1					
	Cyanidin-5-O-β-D-glucoside											C(O)[C@@H]1([C@@H](O)[C@H](O)[C@@H](O)[C@@H](O1)OC3(\C4(/C=C(C(C2(\C=C(C(/O)=C\C=2)\O))=[O+]C(\C=C(/C=3)\[O-])=4)/[O-])))	InChI=1S/C21H20O11/c22-7-16-17(27)18(28)19(29)21(32-16)31-15-5-9(23)4-14-10(15)6-13(26)20(30-14)8-1-2-11(24)12(25)3-8/h1-6,16-19,21-22,27-29H,7H2,(H3-,23,24,25,26)/p-1/t16-,17-,18+,19-,21-/m1/s1					
	Cyanidin-3-O-β-D-galactoside											C(O)[C@@H]1([C@H](O)[C@H](O)[C@@H](O)[C@@H](O1)OC3(\C(C2(\C=C(C(/O)=C\C=2)\O))=[O+]C4(\C(/C=3)=C(/C=C(/C=4)/O)\[O-])))	InChI=1S/C21H20O11/c22-7-16-17(27)18(28)19(29)21(32-16)31-15-6-10-12(25)4-9(23)5-14(10)30-20(15)8-1-2-11(24)13(26)3-8/h1-6,16-19,21-22,27-29H,7H2,(H3-,23,24,25,26)/t16-,17+,18+,19-,21-/m1/s1					
	Anthocyanins Pelargonidin-3-O-β-D-glucoside											C(O)[C@H]1(O[C@H]([C@@H]([C@H]([C@@H]1O)O)O)OC2(\C(=[O+]C3(\C=C(/C=C(C(\C=2)=3)/[O-])\O))C4(\C=C/C(/O)=C\C=4)))	InChI=1S/C21H20O10/c22-8-16-17(26)18(27)19(28)21(31-16)30-15-7-12-13(25)5-11(24)6-14(12)29-20(15)9-1-3-10(23)4-2-9/h1-7,16-19,21-22,26-28H,8H2,(H2-,23,24,25)/t16-,17-,18+,19-,21-/m1/s1					
	Luteolinidin-3-O-β-D-glucoside	23	433.110	5.26	Negative	***	433.1129	$C_{21}H_{21}O_{10}^{+}$	433.1125<br>282.1098<br>271.0600<br>119.0502	433.1140<br>282.1103<br>271.0612<br>119.0502	$C_{21}H_{21}O_{10}^{-}$<br>$C_{14}H_{18}O_{6}$<br>$C_{15}H_{11}O_{5}^{-}$<br>$C_{8}H_{7}O^{-}$	C1=CC(=C(C=C1C2=[O+]C3=C(C=C2)C(=CC(=C3)O)OC4C(C(C(C(O4)CO)O)O)O)O)O	InChI=1S/C21H20O10/c22-8-17-18(26)19(27)20(28)21(31-17)30-16-7-10(23)6-15-11(16)2-4-14(29-15)9-1-3-12(24)13(25)5-9/h1-7,17-22,26-28H,8H2,(H2-,23,24,25)/p+1/t17-,18-,19+,20-,21-/m1/s1	5115bc	8541ab	4182bc	11076a	2627c
	Cyanidin-3-O-rhamnoside											C[C@@H]1O[C@@H](OC2=C([O+]=C3C=C(O)C=C(O)C3=C2)C2=CC(O)=C	InChI=1S/C21H20O10/c1-8-17(26)18(27)19(28)21(29-8)31-16-7-11-13(24)5-10(22)6-15(11)30-					

	 Peonidin-3-O-α-D-arabinopyra-noside											(O)C=C2)[C@H](O)[C@H](O)[C@H]1O<br>COc(c(O)4)cc(cc4)c([o+1]1)c(OC(O3)C(O)C(O)C(O)C3)cc(c(O)2)c(cc(O)c2)1	20(16)9-2-3-12(23)14(25)4-9/h2-8,17-19,21,26-28H,1H3,(H3-,22,23,24,25)/p+1/t8-,17-,18+,19+,21-/m0/s1<br>InChI=1S/C21H20O10/c1-28-16-4-9(2-3-12(16)23)20-17(31-21-19(27)18(26)14(25)8-29-21)7-11-13(24)5-10(22)6-15(11)30-20/h2-7,14,18-19,21,25-27H,8H2,1H3,(H2-,22,23,24)/p+1					
	Derivative of malvidin-3-O-β-D-glucoside	24	475.120	6.4	Negative	***	475.1246	$C_{23}H_{23}O_{11}^-$	271.0600<br>119.0502<br>43.0667	271.0823<br>119.0502<br>43.0189	$C_{12}H_{15}O_7^-$<br>$C_8H_7O^-$<br>$C_2H_3O^-$	COC1(/C=C(/C=C(C(\O)=1)/OC)/C3(C(/O[C@H]2([C@H](O)[C@@H](O)[C@H](O)[C@@H](CO)O2))=C\C4(\C(\[O-])=C/C(/O)=C\C(\[O+]=3)=4)))	InChI=1S/C23H24O12/c1-31-14-3-9(4-15(32-2)18(14)27)22-16(7-11-12(26)5-10(25)6-13(11)33-22)34-23-21(30)20(29)19(28)17(8-24)35-23/h3-7,17,19-21,23-24,28-30H,8H2,1-2H3,(H2-,25,26,27)/t17-,19-,20+,21-,23-/m1/s1	7912ab	10263ab	6502ab	25525a	3287b
	Rutin	25	609.139	4.72	Negative	610.1572	610.1534	$C_{27}H_{30}O_{16}$	301.0327<br>337.1068	301.0357<br>337.0929	$C_{15}H_9O_7^-$<br>$C_{16}H_{17}O_8^-$	C[C@H]1([C@H](O)[C@@H](O)[C@@H](O)[C@@H](O1)OC[C@@H]2([C@@H](O)[C@H](O)[C@@H](O)[C@@H](O2)OC4(\C(=O)C5(/C(/O)=C\C(\O)=C/C(/OC(\C3(/C=C(C(/O)=C\C=3)/O))=4)=5))))	InChI=1S/C27H30O16/c1-8-17(32)20(35)22(37)26(40-8)39-7-15-18(33)21(36)23(38)27(42-15)43-25-19(34)16-13(31)5-10(28)6-14(16)41-24(25)9-2-3-11(29)12(30)4-9/h2-6,8,15,17-18,20-23,26-33,35-38H,7H2,1H3/t8-,15+,17-,18+,20+,21-,22+,23+,26+,27-/m0/s1	8856a	5131a	7060a	12720a	6178a
	Datiscin<br>Kaempferol-3-O-	26	593.143	5.6	Negative	***	594.1585	$C_{27}H_{30}O_{15}$	285.0363<br>284.0271	285.0405<br>284.0332	$C_{15}H_9O_6^-$<br>$C_{15}H_8O_6^{2-}$	CC1C(C(C(C(O1)OCC2C(C(C(C(O2)OC3=C(OC4=CC(=CC(=C4C3=O)O)O)C5=CC=CC=C5O)O)O)O)O)O)O<br>C[C@@H]5(O[C@@H](OC[C@H]4(O[C@@H](OC2(\C(C3	InChI=1S/C27H30O15/c1-9-17(31)20(34)22(36)26(39-9)38-8-15-18(32)21(35)23(37)27(41-15)42-25-19(33)16-13(30)6-10(28)7-14(16)40-24(25)11-4-2-3-5-12(11)29/h2-7,9,15,17-18,20-23,26-32,34-37H,8H2,1H3/t9-,15+,17-,18+,20+,21-,22+,23+,26+,27-/m0/s1<br>InChI=1S/C27H30O15/c1-9-17(31)20(34)22(36)26(39-9)38-8-15-	12415a	10612a	7922a	17802a	8287a

	Rutinoside											(/C(/OC(\C1(/C=C\C(\O)=C/C=1))=2)=C\C(\O)=C/C(/O)=3))=O))[C@@H]([C@H]([C@@H]4O)O)O))[C@@H]([C@@H]([C@H]5O)O)O)	18(32)21(35)23(37)27(41-15)42-25-19(33)16-13(30)6-12(29)7-14(16)40-24(25)10-2-4-11(28)5-3-10/h2-7,9,15,17-18,20-23,26-32,34-37H,8H2,1H3/t9-,15+,17-,18+,20+,21-,22+,23+,26+,27-/m0/s1					
	Kaempferol-3-glucoside-7-rhamnoside											C[C@@H]5(O[C@@H](OC4(\C=C3(C(\C(=O)C(/O[C@@H]1(O[C@@H]([C@H]([C@@H]([C@H]1O)O)O)CO))=C(C2(\C=C/C(/O)=C\C=2))\O3)=C(/C=4)\O)))[C@@H]([C@@H]([C@H]5O)O)O)	InChI=1S/C27H30O15/c1-9-17(31)20(34)22(36)26(38-9)39-12-6-13(30)16-14(7-12)40-24(10-2-4-11(29)5-3-10)25(19(16)33)42-27-23(37)21(35)18(32)15(8-28)41-27/h2-7,9,15,17-18,20-23,26-32,34-37H,8H2,1H3/t9-,15+,17-,18+,20+,21-,22+,23+,26-,27-/m0/s1					
	Kaempferol 3-o-rhamnoside-7-o-glucoside											C(O)[C@@H]5([C@@H](O)[C@H](O)[C@@H](O)[C@H](OC[C@@H]4([C@@H](O)[C@H](O)[C@@H](O)[C@H](OC2(\C=C(C3(\C(=O)C(/C1(\C=C/C(/O)=C\C=1))=C/OC(/C=2)=3))\O))O4))O5)	InChI=1S/C27H30O15/c28-7-16-20(32)22(34)24(36)26(41-16)39-9-17-21(33)23(35)25(37)27(42-17)40-12-5-14(30)18-15(6-12)38-8-13(19(18)31)10-1-3-11(29)4-2-10/h1-6,8,16-17,20-30,32-37H,7,9H2/t16-,17-,20-,21-,22+,23+,24-,25-,26-,27-/m1/s1					
	Genistin 7-gentiobioside											C(O)[C@@H]5([C@@H](O)[C@H](O)[C@@H](O)[C@H](OC[C@@H]4([C@@H](O)[C@H](O)[C@@H](O)[C@H](OC2(\C=C(C3(\C(=O)C(/C1(\C=C/C(/O)=C\C=1))=C/OC(/C=2)=3))\O))O4))O5)	InChI=1S/C27H30O15/c28-7-16-20(32)22(34)24(36)26(41-16)39-9-17-21(33)23(35)25(37)27(42-17)40-12-5-14(30)18-15(6-12)38-8-13(19(18)31)10-1-3-11(29)4-2-10/h1-6,8,16-17,20-30,32-37H,7,9H2/t16-,17-,20-,21-,22+,23+,24-,25-,26-,27-/m1/s1					
												C[C@@H]5(O[C@@H](O[C@@H]1([C@H]([C@@H]([C	InChI=1S/C27H30O15/c1-9-20(33)22(35)24(37)26(38-9)42-25-					

	Luteolin 7-o-neohesperi-doside											@@H](CO)O[C@H]1OC4(\C=C3(C(\C(\C=C(C2(\C=C(C(/O)=C\C=2)\O))/O3)=O)=C(/C=4)\O)))O)O))[C@@H]([C@@H]([C@H]5O)O)O)	23(36)21(34)18(8-28)41-27(25)39-11-5-14(31)19-15(32)7-16(40-17(19)6-11)10-2-3-12(29)13(30)4-10/h2-7,9,18,20-31,33-37H,8H2,1H3/t9-,18+,20-,21+,22+,23-,24+,25+,26-,27+/m0/s1					
	Apigenin-7-o-gentiobioside											C5(/C=C(\C=C/C(/C1(/OC2(/C=C(/C=C(C(\C(=O)\C=1)=2)/O)/O[C@@H]4(O[C@H](CO[C@@H]3(O[C@H](CO)[C@@H](O)[C@H](O)[C@@H](O)3))[C@@H](O)[C@H](O)[C@@H](O)4))))=5)/O)	InChI=1S/C27H30O15/c28-8-17-20(32)22(34)24(36)26(41-17)38-9-18-21(33)23(35)25(37)27(42-18)39-12-5-13(30)19-14(31)7-15(40-16(19)6-12)10-1-3-11(29)4-2-10/h1-7,17-18,20-30,32-37H,8-9H2/t17-,18-,20-,21-,22+,23+,24-,25-,26-,27-/m1/s1					
	Isovitexin 2-O-β-D-glucoside											C5(/C=C(\C=C/C(/C1(/OC4(\C(\C(=O)\C=1)=C(C(\[C@@H]3(O[C@H](CO)[C@@H](O)[C@H](O)[C@@H](O[C@@H]2(O[C@H](CO)[C@@H](O)[C@H](O)[C@@H](O)2))3))=C(/C=4)/[O-])\O)))=5)/O)	InChI=1S/C27H30O15/c28-7-15-20(34)23(37)26(42-27-24(38)22(36)19(33)16(8-29)41-27)25(40-15)18-12(32)6-14-17(21(18)35)11(31)5-13(39-14)9-1-3-10(30)4-2-9/h1-6,15-16,19-20,22-30,32-38H,7-8H2/p-1/t15-,16-,19-,20-,22+,23+,24-,25+,26-,27+/m1/s1					
	Isovitexin 7-o-galactoside											C5(/C=C(\C=C/C(/C1(/OC3(\C(\C(=O)\C=1)=C(C(\[C@@H]2(O[C@H](CO)[C@@H](O)[C@H](O)[C@@H](O)2))=C(/C=3)/O[C@@H]4([C@@H]([C@H]([C@@H](O)[C@H](O4)CO)O)O))\O)))=5)/O)	InChI=1S/C27H30O15/c28-7-15-19(32)22(35)24(37)26(40-15)18-14(41-27-25(38)23(36)20(33)16(8-29)42-27)6-13-17(21(18)34)11(31)5-12(39-13)9-1-3-10(30)4-2-9/h1-6,15-16,19-20,22-30,32-38H,7-8H2/t15-,16-,19-,20+,22+,23+,24-,25-,26+,27+/m1/s1					
	Isovitexin 7-O-glucoside											C5(/C=C(\C=C/C(/C1(/OC3(\C(\C(=O)\C=1)=C(C(\[C@@H]2(O[C@H](CO)[C@@H](O)[C@H](O)[	InChI=1S/C27H30O15/c28-7-15-19(32)22(35)24(37)26(40-15)18-14(41-27-25(38)23(36)20(33)16(8-29)42-27)6-13-17(21(18)34)11(31)5-					

												C@@H](O)2))=C(/C=3)/O[C@@H]4(O[C@H](CO)[C@@H](O)[C@H](O)[C@@H](O)4))\O)))=5)/O)	12(39-13)9-1-3-10(30)4-2-9/h1-6,15-16,19-20,22-30,32-38H,7-8H2/t15-,16-,19-,20-,22+,23+,24-,25-,26+,27-/m1/s1					
	Rhamnosylisoorientin											C[C@H]1([C@H](O)[C@@H](O)[C@@H](O)C(O1)O[C@H]5([C@H](C3(/C(/[O-])=C\C4(\OC(/C2(\C=C(C(/O)=C\C=2)\O))=C/C(C(/C(/O)=3)=4)=O)))O[C@H](CO)[C@@H](O)[C@H](O)5))	InChI=1S/C27H30O15/c1-8-19(33)22(36)24(38)27(39-8)42-26-23(37)20(34)16(7-28)41-25(26)18-13(32)6-15-17(21(18)35)12(31)5-14(40-15)9-2-3-10(29)11(30)4-9/h2-6,8,16,19-20,22-30,32-38H,7H2,1H3/p-1/t8-,16+,19-,20+,22+,23-,24+,25-,26+,27?/m0/s1					
	Vitexin 2-O-β-D-glucoside											C([C@@H]4([C@H]([C@@H]([C@H]([C@H](C2(/C3(/OC(/C1(\C=C/C(/O)=C\C=1))=C/C(C(/C(/O)=C\C(\[O-])=2)=3)=O)))O4)O[C@H]5([C@@H]([C@H]([C@@H]([C@H](O5)CO)O)O)O))O)O))O	InChI=1S/C27H30O15/c28-7-15-20(35)22(37)26(42-27-23(38)21(36)19(34)16(8-29)41-27)25(40-15)18-12(32)5-11(31)17-13(33)6-14(39-24(17)18)9-1-3-10(30)4-2-9/h1-6,15-16,19-23,25-32,34-38H,7-8H2/p-1/t15-,16-,19-,20-,21+,22+,23-,25+,26-,27+/m1/s1					
	Vicenin-2											C(O)[C@@H]5([C@@H](O)[C@H](O)[C@@H](O)[C@H](C3(/C(/[O-])=C([C@@H]1(O[C@H](CO)[C@@H](O)[C@H](O)[C@@H](O)1))\C4(\OC(/C2(\C=C/C(/O)=C\C=2))=C/C(=O)C(/C(/O)=3)=4)))O5)	InChI=1S/C27H30O15/c28-6-12-17(32)21(36)23(38)26(41-12)15-19(34)14-10(31)5-11(8-1-3-9(30)4-2-8)40-25(14)16(20(15)35)27-24(39)22(37)18(33)13(7-29)42-27/h1-5,12-13,17-18,21-24,26-30,32-39H,6-7H2/p-1/t12-,13-,17-,18-,21+,22+,23-,24-,26+,27+/m1/s1					
**Terpenoids**	Ω-carboxymenaquinone-4	27	473.275	18.03	Negative	***	474.277	$C_{31}H_{38}O_4$	205.0598	205.0539	$C_{14}H_{21}O^-$	CC(/CC/C=C(C)/CC/C=C(C)/C(=O)[O-])=C\CCC(\C)=C/CC1(\C(=O)C2(/C=C\C=C/C(/C(=O)C(/C)=1)=2))	InChI=1S/C31H38O4/c1-21(11-8-12-22(2)15-10-16-24(4)31(34)35)13-9-14-23(3)19-20-26-25(5)29(32)27-17-6-7-18-28(27)30(26)33/h6-7,12-13,16-19H,8-11,14-15,20H2,1-	1667c	14798a	14718a	12107ab	11427b

													5H3,(H,34,35)/p-1/b21-13+,22-12+,23-19+,24-16+					
	4-imino-demethoxy-ubiquinone	28	559.467	17.43	Negative	***	559.4389	$C_{38}H_{57}NO_2$	297.2312 280.2331	297.2330 280.2282	$C_{18}H_{31}O_2^-$ $C_{17}H_{30}NO_2^-$	CC(C)=CCCC(\C)=C/CCC(\C)=C/CCC(\C)=C/CCC(\C)=C/CCC(\C)=C/CC1(\C(C(/OC)=C\C(C(/C)=1)=O)=N)	InChI=1S/C38H57NO2/c1-28(2)15-10-16-29(3)17-11-18-30(4)19-12-20-31(5)21-13-22-32(6)23-14-24-33(7)25-26-35-34(8)36(40)27-37(41-9)38(35)39/h15,17,19,21,23,25,27,39H,10-14,16,18,20,22,24,26H2,1-9H3/b29-17+,30-19+,31-21+,32-23+,33-25+,39-38?	4770b	6039ab	4516b	8619a	4276b
	Derivative of menaquinol-6	29			Negative	***	560.4593	$C_{39}H_{60}O_2$				CC(C)=CCCC(\C)=C/CCC(\C)=C/CCC(\C)=C/CCC(\C)=C/CCC(\C)=C/CC1(/C(\C)=C(C2(/C=C\C=C/C(/C(/O)=1)=2))/O)	InChI=1S/C41H58O2/c1-30(2)16-11-17-31(3)18-12-19-32(4)20-13-21-33(5)22-14-23-34(6)24-15-25-35(7)28-29-37-36(8)40(42)38-26-9-10-27-39(38)41(37)43/h9-10,16,18,20,22,24,26-28,42-43H,11-15,17,19,21,23,25,29H2,1-8H3/b31-18+,32-20+,33-22+,34-24+,35-28+	2517bc	3877a	1425c	2805ab	1425c
	4-imino-demethoxy-ubiquinone Menaquinol-6 derivative	30	431.215	14.36	Negative	***	***	***	280.2331 152.9945	280.2282 152.9969	$C_{17}H_{30}NO_2^-$ $C_3H_6O_5^{3-}$	CC(C)=CCCC(\C)=C/CCC(\C)=C/CCC(\C)=C/CCC(\C)=C/CCC(\C)=C/CC1(\C(C(/OC)=C\C(C(/C)=1)=O)=N)	InChI=1S/C38H57NO2/c1-28(2)15-10-16-29(3)17-11-18-30(4)19-12-20-31(5)21-13-22-32(6)23-14-24-33(7)25-26-35-34(8)36(40)27-37(41-9)38(35)39/h15,17,19,21,23,25,27,39H,10-14,16,18,20,22,24,26H2,1-9H3/b29-17+,30-19+,31-21+,32-23+,33-25+,39-38?	1197c	34993bc	55579abc	109061ab	118330a
		31	433.223	15.47	Negative	***	***	***				CC(C)=CCCC(\C)=C/CCC(\C)=C/CCC(\C)=C/CCC(\C)=C/CCC(\C)=C/CC1(/C(\C)=C(C2(/C=C\C=C/C(/C(/O)=1)=2))/O)	InChI=1S/C41H58O2/c1-30(2)16-11-17-31(3)18-12-19-32(4)20-13-21-33(5)22-14-23-34(6)24-15-25-35(7)28-29-37-36(8)40(42)38-26-9-10-27-39(38)41(37)43/h9-10,16,18,20,22,24,26-28,42-43H,11-15,17,19,21,23,25,29H2,1-8H3/b31-18+,32-20+,33-22+,34-24+,35-28+	9618c	188415b	4435c	397843a	227555b
	Fucoxanthi-nol	32	616.407	12.62	Positive	***	616.4128	$C_{40}H_{56}O_5$	514.3528 313.3120 183.1204 183.1001	514.3436 313.3101 183.1380 183.1016	$C_{35}H_{46}O_3^{2+}$ $C_{20}H_{41}O_2^+$ $C_{11}H_{19}O_2^+$ $C_{10}H_{15}O_3^+$	CC(/C=C/C=C(C)/C=C=C1([C@@](O)(C)C[C@@H](O)CC(C)(C)1))=C\C=C\C	InChI=1S/C40H56O5/c1-28(17-13-18-30(3)21-22-35-36(5,6)23-32(41)25-38(35,9)44)15-11-12-16-	1125c	5616bc	7083b	13226a	7848ab

									122.0848<br>102.0708	122.0727<br>102.0670	$C_8H_{10}O^{2+}$<br>$C_5H_{10}O_2^{2+}$	=C(C)\C=C\C=C(C)\C(=O)C[C@]23(C(C)(C)C[C@H](O)C[C@@](C)(O2)3)	29(2)19-14-20-31(4)34(43)27-40-37(7,8)24-33(42)26-39(40,10)45-40/h11-21,32-33,41-42,44H,23-27H2,1-10H3/b12-11+,17-13+,19-14+,28-15+,29-16+,30-18+,31-20+/t22?,32-,33-,38+,39+,40-/m0/s1					
	4,4'-diaponeuros-porenal<br><br>8'-apo-β-carotenal<br><br>apo-8'-lycopenal<br><br>4-hydroxy-4,4'-diapo-lycopene	33	417.303	15.71	Positive	***	416.3079	$C_{30}H_{40}O$	417.3035<br>337.3195<br>318.3003<br>263.2755<br>151.1026<br>67.0690	417.3152<br>337.3101<br>318.2912<br>263.2733<br>151.1117<br>67.0542	$C_{30}H_{41}O^+$<br>$C_{22}H_{41}O_2^+$<br>$C_{22}H_{38}O^{2+}$<br>$C_{19}H_{35}^+$<br>$C_{10}H_{15}O^+$<br>$C_5H_7O^+$	CC(C)=CCCC(\C)=C/C=C/C(\C)=C/C=C/C=C(C)/C=C/C=C(C)/C=C/C=C(C)/C=O<br><br>CC(/C=C/C=C(C)/C=C/C1(/C(CCCC(/C)=1)(C)C))=C\C=C\C=C(C)\C=C\C=C(C)\C=O<br><br>CC(C)=CCCC(\C)=C/C=C/C(\C)=C/C=C/C(\C)=C/C=C/C=C(C)/C=C/C=C(C)/C=O<br><br>CC(C)=C/C=C/C(\C)=C/C=C/C(\C)=C/C=C/C=C(C)/C=C/C=C(C)/C=C/C=C(C)/CO	InChI=1S/C30H40O/c1-25(2)14-10-17-28(5)20-11-18-26(3)15-8-9-16-27(4)19-12-21-29(6)22-13-23-30(7)24-31/h8-9,11-16,18-24H,10,17H2,1-7H3/b9-8+,18-11+,19-12+,22-13+,26-15+,27-16+,28-20+,29-21+,30-23+<br><br>InChI=1S/C30H40O/c1-24(13-8-9-14-25(2)16-11-18-27(4)23-31)15-10-17-26(3)20-21-29-28(5)19-12-22-30(29,6)7/h8-11,13-18,20-21,23H,12,19,22H2,1-7H3/b9-8+,15-10+,16-11+,21-20+,24-13+,25-14+,26-17+,27-18+<br><br>InChI=1S/C30H40O/c1-25(2)14-10-17-28(5)19-12-21-29(6)20-11-18-26(3)15-8-9-16-27(4)22-13-23-30(7)24-31/h8-9,11-16,18-24H,10,17H2,1-7H3/b9-8+,18-11+,21-12+,22-13+,26-15+,27-16+,28-19+,29-20+,30-23+<br><br>InChI=1S/C30H40O/c1-25(2)14-10-17-28(5)20-11-18-26(3)15-8-9-16-27(4)19-12-21-29(6)22-13-23-30(7)24-31/h8-23,31H,24H2,1-7H3/b9-8+,17-10+,18-11+,19-12+,22-13+,26-15+,27-16+,28-20+,29-21+,30-23+	9744b	33258a	21828ab	22693ab	28704ab

	4,4'-diapo-neurosporen-1-ol<br><br>8'-apo-β-carotenol	34	418.316	15.39	Positive	***	418.3236	$C_{30}H_{42}O$	337.3508<br>263.2755<br>81.0022	337.3465<br>263.2733<br>81.0699	$C_{23}H_{45}O^+$<br>$C_{19}H_{35}^+$<br>$C_6H_9^+$	CC(C)=CCCC(\C)=C/C=C/C(\C)=C/C=C/C=C(C)/C=C/C=C(C)/C=C/C=C(C)/CO<br><br>CC(/C=C/C=C(C)/C=C/C1(/C(C)(C)CCCC(/C)=1))=C\C=C\C=C(C)\C=C\C=C(C)\CO	InChI=1S/C30H42O/c1-25(2)14-10-17-28(5)20-11-18-26(3)15-8-9-16-27(4)19-12-21-29(6)22-13-23-30(7)24-31/h8-9,11-16,18-23,31H,10,17,24H2,1-7H3/b9-8+,18-11+,19-12+,22-13+,26-15+,27-16+,28-20+,29-21+,30-23+<br><br>InChI=1S/C30H42O/c1-24(13-8-9-14-25(2)16-11-18-27(4)23-31)15-10-17-26(3)20-21-29-28(5)19-12-22-30(29,6)7/h8-11,13-18,20-21,31H,12,19,22-23H2,1-7H3/b9-8+,15-10+,16-11+,21-20+,24-13+,25-14+,26-17+,27-18+	5383ab	7527ab	7157ab	12007a	696b
	Derivative	35	415.275	14.48	Positive	***	***	***	317.2824<br>316.2702<br>135.1185<br>99.0026<br>67.0690	317.2839<br>316.2755<br>135.1168<br>99.0804<br>67.0542	$C_{22}H_{37}O^+$<br>$C_{22}H_{36}O^+$<br>$C_{10}H_{15}^+$<br>$C_6H_{11}O^+$<br>$C_5H_7O^+$			3773b	6509b	7232ab	13061ab	17529a
	4,4'-diapo-neurosporen-1,1-diol	36	435.309	15.46	Positive	***	434.3185	$C_{30}H_{42}O_2$	435.3114<br>337.3195<br>280.2745	435.3258<br>337.3101<br>280.2755	$C_{30}H_{43}O_2^+$<br>$C_{22}H_{41}O_2^+$<br>$C_{19}H_{36}O_2^+$	CC(C)=CCCC(\C)=C/C=C/C(\C)=C/C=C/C=C(C)/C=C/C=C(C)/C=C/C=C(C)/C(O)O	InChI=1S/C30H42O2/c1-24(2)14-10-17-27(5)20-11-18-25(3)15-8-9-16-26(4)19-12-21-28(6)22-13-23-29(7)30(31)32/h8-9,11-16,18-23,30-32H,10,17H2,1-7H3/b9-8+,18-11+,19-12+,22-13+,25-15+,26-16+,27-20+,28-21+,29-23+	11247b	9062b	5276b	20988a	7055b
	All-trans-3,4-didehydro-lycopene	37	534.429	13.65	Positive	***	534.4226	$C_{40}H_{54}$	263.2755<br>245.2218<br>179.1751<br>81.0874	263.2733<br>245.2264<br>179.1794<br>81.0699	$C_{19}H_{35}^+$<br>$C_{18}H_{29}^+$<br>$C_{13}H_{23}^+$<br>$C_6H_9^+$	CC(C)=C/C=C/C(\C)=C/C=C/C(\C)=C/C=C/C(\C)=C/C=C/C=C(C)/C=C/C=C(C)/C=C/C=C(C)/CCC=C(C)C	InChI=1S/C40H54/c1-33(2)19-13-23-37(7)27-17-31-39(9)29-15-25-35(5)21-11-12-22-36(6)26-16-30-40(10)32-18-28-38(8)24-14-20-34(3)4/h11-13,15-23,25-32H,14,24H2,1-10H3/b12-11+,23-13+,25-15+,26-16+,31-17+,32-18+,35-21+,36-22+,37-27+,38-28+,39-29+,40-30+	20063bc	39634ab	21145bc	54265a	6482c
		38	535.401	13.64	Positive	***	***	***	263.2755<br>245.2218<br>179.1751<br>81.0874	263.2733<br>245.2264<br>179.1794<br>81.0699	$C_{19}H_{35}^+$<br>$C_{18}H_{29}^+$<br>$C_{13}H_{23}^+$<br>$C_6H_9^+$			743b	1531b	5909b	19405a	3056b
	Derivative	39	520.418	12.75	Positive	***	***	***	453.3598<br>353.3291	453.3516<br>353.3203	$C_{34}H_{45}^+$<br>$C_{26}H_{41}^+$			12722a	9148a	16067a	25445a	7848a
	2-hexaprenyl-6-methoxy-phenol	40	532.416	12.75	Positive	***	532.4280	$C_{37}H_{56}O_2$	353.3291<br>179.1751	353.3203<br>179.1794	$C_{26}H_{41}^+$<br>$C_{11}H_{15}O^+$	CC(C)=CCCC(\C)=C/CCC(\C)=C/CCC(\C)=C/CCC(\C)=C/CCC(\C)=C/CC1(/C=C\C=C(C(/O)=1)\OC)	InChI=1S/C37H56O2/c1-29(2)15-9-16-30(3)17-10-18-31(4)19-11-20-32(5)21-12-22-33(6)23-13-24-34(7)27-28-35-25-14-26-36(39-	31548a	21610a	58841a	55124a	25543a

													8)37(35)38/h14-15,17,19,21,23,25-27,38H,9-13,16,18,20,22,24,28H2,1-8H3/b30-17+,31-19+,32-21+,33-23+,34-27+					
	Methano-phenazine	41	539.390	13.65	Positive	***	538.3923	$C_{37}H_{50}N_2O$	539.3913<br>355.3604<br>337.3195<br>263.2755<br>245.2534<br>184.0922	539.3996<br>355.3683<br>337.3213<br>263.2733<br>245.2587<br>184.1000	$C_{37}H_{51}N_2O^+$<br>$C_{22}H_{47}N_2O^+$<br>$C_{21}H_{41}N_2O^+$<br>$C_{19}H_{35}^+$<br>$C_{14}H_{33}N_2O^+$<br>$C_{12}H_{12}N_2$	CC(C)=CCCC(\C)=C/CCC(\C)=C/CCC(\C)=C/CCC(CCOC2(\C=C/C3(/N=C1(\C=C/C=C\C/1=N\C(/C=2)=3))))C	InChI=1S/C37H50N2O/c1-28(2)13-9-14-29(3)15-10-16-30(4)17-11-18-31(5)19-12-20-32(6)25-26-40-33-23-24-36-37(27-33)39-35-22-8-7-21-34(35)38-36/h7-8,13,15,17,19,21-24,27,32H,9-12,14,16,18,20,25-26H2,1-6H3/b29-15+,30-17+,31-19+	51773bc	98094ab	53799bc	115828a	17443c
	Methano-phenazine derivative	42	537.373	12.75	Positive	***	538.3923	$C_{37}H_{50}N_2O$	537.3753<br>183.0871	537.3839<br>183.0839	$C_{37}H_{49}N_2O^+$<br>$C_{12}H_{11}N_2^+$			66347a	57327a	146539a	108571a	60307a
	2-methoxy-6-all trans-nonaprenyl-1,4-benzoquinol	43	752.584	13.57	Positive	***	752.6107	$C_{52}H_{80}O_3$	589.4676<br>277.2592<br>163.1175<br>121.0974	589.4615<br>277.2526<br>163.1117<br>121.1012	$C_{40}H_{61}O_3^+$<br>$C_{19}H_{33}O^+$<br>$C_{11}H_{15}O^+$<br>$C_9H_{13}^+$	CC(C)=CCCC(\C)=C/CCC(\C)=C/CCC(\C)=C/CCC(\C)=C/CCC(\C)=C/CCC(\C)=C/CCC(\C)=C/CCC(\C)=C/CC1(\C(\O)=C(/C=C(\C=1)/O)\OC)	InChI=1S/C52H80O3/c1-40(2)20-12-21-41(3)22-13-23-42(4)24-14-25-43(5)26-15-27-44(6)28-16-29-45(7)30-17-31-46(8)32-18-33-47(9)34-19-35-48(10)36-37-49-38-50(53)39-51(55-11)52(49)54/h20,22,24,26,28,30,32,34,36,38-39,53-54H,12-19,21,23,25,27,29,31,33,35,37H2,1-11H3/b41-22+,42-24+,43-26+,44-28+,45-30+,46-32+,47-34+,48-36+	6640b	232749a	288551a	266959a	301675a
	Derivative of 2-methoxy-6-all trans-nonaprenyl-1,4-benzoquinol	44	708.562	13.60	Positive	***	***	***	621.5035<br>431.3211<br>121.0974	621.5030<br>431.3308<br>121.1012	$C_{45}H_{65}O^+$<br>$C_{31}H_{43}O^+$<br>$C_9H_{13}^+$			34287b	347244a	426550a	396494a	412364a
	ubiquinol-9	45	796.619	13.53	Positive	***	796.6370	$C_{54}H_{84}O_4$	779.6335<br>175.0936<br>131.1057	779.6337<br>175.0965<br>131.1067	$C_{54}H_{83}O_3^+$<br>$C_8H_{15}O_4^+$<br>$C_7H_{15}O_2^+$	CC(C)=CCCC(\C)=C/CCC(\C)=C/CCC(\C)=C/CCC(\C)=C/CCC(\C)=C/CCC(\C)=C/CCC(\C)=C/CCC(\C)=C/CC1(\C(\O)=C(C(\OC)=C(C(/C)=1)\O)\OC)	InChI=1S/C54H84O4/c1-40(2)22-14-23-41(3)24-15-25-42(4)26-16-27-43(5)28-17-29-44(6)30-18-31-45(7)32-19-33-46(8)34-20-35-47(9)36-21-37-48(10)38-39-50-49(11)51(55)53(57-12)54(58-13)52(50)56/h22,24,26,28,30,32,34,36,38,55-56H,14-21,23,25,27,29,31,33,35,37,39H2,1-13H3/b41-24+,42-26+,43-28+,44-30+,45-32+,46-34+,47-36+,48-38+	2201b	146765a	192982a	173175a	194954a
	Isopentenyl-dehydrorho-dopin	46	620.498	13.70	Positive	***	620.4957	$C_{45}H_{64}O$	620.4885<br>227.2592<br>177.1642	620.4946<br>227.2526<br>177.1638	$C_{45}H_{64}O^{2+}$<br>$C_{19}H_{33}O^+$<br>$C_{13}H_{21}^+$	CC(C)=CCCC(\C)=C/C=C/C(\C)=C/C=C/C(\C)=C/C=C/C=C(C)/C=C/C=C(C)/C=C/C=C(C)/C=C/[	InChI=1S/C45H64O/c1-36(2)20-15-23-40(7)26-18-29-41(8)27-16-24-38(5)21-13-14-22-39(6)25-17-28-42(9)30-	94217b	697620a	578804a	573162a	612053a
	Isopentenyl-dehydrorh-	47	884.676	13.45	Positive	***	***	***	309.2297<br>233.2234	309.2213<br>233.2264	$C_{22}H_{29}O^+$<br>$C_{17}H_{29}O^+$			615b	20631a	29899a	28002a	27889a

	odopin derivative								177.1642<br>119.0752	177.1638<br>119.0855	$C_{13}H_{21}^{+}$<br>$C_{9}H_{11}^{+}$	C@@H](C(C)(C)O)CC=C(C)C	19-31-43(10)33-35-44(45(11,12)46)34-32-37(3)4/h13-14,16-22,24-33,35,44,46H,15,23,34H2,1-12H3/b14-13+,24-16+,25-17+,29-18+,30-19+,35-33+,38-21+,39-22+,40-26+,41-27+,42-28+,43-31+/t44-/m0/s1					
		48	664.528	13.65	Positive	***	***	***	499.4002<br>129.1272<br>89.0703	499.3934<br>129.1274<br>89.0961	$C_{36}H_{31}O^{+}$<br>$C_{13}H_{21}^{+}$<br>$C_{5}H_{13}O^{+}$			71318b	620746a	486803a	476005a	488965a
	Bixin dimethyl ester	49	409.243	4.55	Positive	***	408.2301	$C_{26}H_{32}O_{4}$	409.2423<br>387.2517<br>189.1635	409.2373<br>387.2530<br>189.1638	$C_{26}H_{33}O_{4}^{+}$<br>$C_{24}H_{35}O_{4}^{+}$<br>$C_{14}H_{21}^{+}$	CC(/C=C/C=C(C)/C=C/C(OC)=O)=C\C=C\C=C(C)\C=C\C=C(C)\C=C\C(OC)=O	InChI=1S/C26H32O4/c1-21(13-9-15-23(3)17-19-25(27)29-5)11-7-8-12-22(2)14-10-16-24(4)18-20-26(28)30-6/h7-20H,1-6H3/b8-7+,13-9+,14-10+,19-17+,20-18+,21-11+,22-12+,23-15+,24-16+	5379a	7332a	6579a	7819a	6025a
	4,4'-diapo-lycopene-4-oate	50	430.287	4.10	Positive	***	430.2872	$C_{30}H_{38}O_{2}$	325.2162<br>251.2056<br>127.0729	325.2128<br>251.2006<br>127.0754	$C_{22}H_{29}O_{2}^{+}$<br>$C_{16}H_{27}O_{2}^{+}$<br>$C_{7}H_{11}O_{2}^{+}$	CC(C)=C/C=C/C(\C)=C/C=C/C(\C)=C/C=C/C=C(C)/C=C/C=C(C)/C=C/C=C(C)/C([O-])=O	InChI=1S/C30H38O2/c1-24(2)14-10-17-27(5)20-11-18-25(3)15-8-9-16-26(4)19-12-21-28(6)22-13-23-29(7)30(31)32/h8-23H,1-7H3,(H,31,32)/p-1/b9-8+,17-10+,18-11+,19-12+,22-13+,25-15+,26-16+,27-20+,28-21+,29-23+	610b	6060a	2401ab	3341ab	1403ab
	oat triterpenoid saponin	51	1093.58	8.88	Positive	***	1093.546	$C_{55}H_{83}NO_{21}$	1023.509<br>1007.475<br>977.4956<br>116.1053	1023.516<br>1007.485<br>977.4952<br>116.1070	$C_{52}H_{79}O_{20}^{+}$<br>$C_{51}H_{75}O_{20}^{+}$<br>$C_{47}H_{77}O_{21}^{+}$<br>$C_{6}H_{14}NO^{+}$	C[C@]2(C=O)([C@@H](OC(=O)C1(\C(\NC)=C/C=C\C=1))C[C@@]4(C)([C@@H](C2)[C@@]35(O[C@@H]3C[C@H]6([C@](C)([C@](C)(C[C@H](O)4)5)CC[C@@H]%10([C@@](C)6CC[C@H](O[C@@H]9(OC[C@H](O[C@@H]7(O[C@H](CO)[C@@H](O)[C@H](O)[C@@H](O)7))[C@H](O)[C@@H](O[C@@H]8(O[C@H](CO)[C@@H](O)[C@H](O)[C@@H](O)8))9))[C@](CO)(C)%10))))	InChI=1S/C55H83NO21/c1-49(23-59)17-32-51(3,19-36(49)74-45(69)25-10-8-9-11-26(25)56-7)33(61)18-54(6)53(5)15-12-30-50(2,31(53)16-35-55(32,54)77-35)14-13-34(52(30,4)24-60)75-48-44(76-47-43(68)41(66)38(63)28(21-58)72-47)39(64)29(22-70-48)73-46-42(67)40(65)37(62)27(20-57)71-46/h8-11,23,27-44,46-48,56-58,60-68H,12-22,24H2,1-7H3/t27-,28-,29+,30-,31-,32-,33+,34+,35-,36+,37-,38-,39+,40+,41+,42-,43-,44-,46+,47+,48+,49+,50+,51+,52+,53-,54+,55-/m1/s1	334b	5430b	45088a	24175ab	18271ab

**Oligosaccharides**	Maltopenta-ose derivative	52	537.321	14.41	Negative	537.321	537.2036	$C_{19}H_{37}O_{17}^-$	282.089	282.0962	$C_{10}H_{18}O_9^{2-}$	C([C@H]5(O[C@H](O[C@@H]4([C@H](O[C@H](O[C@@H]1([C@H](O[C@@H]([C@@H]([C@H]1O)O)O[C@@H]2([C@H](O[C@@H]([C@@H]([C@H]2O)O)O[C@@H]3([C@H](OC([C@@H]([C@H]3O)O)O)CO))CO))CO))[C@@H]([C@H]4O)O)CO))[C@@H]([C@H]([C@@H]5O)O)O))O	InChI=1S/C30H52O26/c31-1-6-11(36)12(37)18(43)27(49-6)54-23-8(3-33)51-29(20(45)14(23)39)56-25-10(5-35)52-30(21(46)16(25)41)55-24-9(4-34)50-28(19(44)15(24)40)53-22-7(2-32)48-26(47)17(42)13(22)38/h6-47H,1-5H2/t6-,7-,8-,9-,10-,11-,12+,13-,14-,15-,16-,17-,18-,19-,20-,21-,22-,23-,24-,25-,26?,27-,28-,29-,30-/m1/s1	7707bc	15163a	10442ab	13790ab	3479c
		53	699.371	12.92	Negative	699.371	699.3081	$C_{30}H_{51}O_{18}^-$	255.2312	255.2330	$C_{16}H_{31}O_2^-$			8278bc	10237b	8050bc	15818a	4038bc
**Organosulfates**	Dodecyl sulfate	54	265.147	14.37	Negative	***	266.1552	$C_{12}H_{26}O_4S$	265.1483<br>185.1888<br>79.9586<br>96.9639	265.1479<br>185.1911<br>79.9579<br>96.9601	$C_{12}H_{25}O_4S^-$<br>$C_{12}H_{25}O^-$<br>O_3S^{-2}<br>HO_4S^-	CCCCCCCCCCCCOS([O-])(=O)=O	InChI=1S/C12H26O4S/c1-2-3-4-5-6-7-8-9-10-11-12-16-17(13,14)15/h2-12H2,1H3,(H,13,14,15)/p-1	664b	33273a	36116a	22823a	21551a
**Galactolipids**	1-16:0-2-18:1-digalactosyl-diacyl-glycerol<br><br>1-18:1-2-16:0-digalactosyl-diacyl-glycerol	55	918.598	5.72	Positive	***	918.628	$C_{49}H_{90}O_{15}$	755.5129<br>414.4031<br>163.0601	755.5151<br>414.4073<br>163.0965	$C_{38}H_{75}O_{14}^+$<br>$C_{26}H_{54}O_3$<br>$C_7H_{15}O_4^+$	CCCCCCCC\C=C/CCCCCCCC(OC(COC2(OC(COC1(OC(CO)C(O)C(O)C(O)1))C(O)C(O)C(O)2))COC(=O)CCCCCCCCCCCCCCC)=O<br><br>CCCCCCCC\C=C/CCCCCCCC(OC[C@H](CO[C@@H]2(O[C@H](CO[C@@H]1(O[C@H](CO)[C@H](O)[C@H](O)[C@@H](O)1))[C@H](O)[C@H](O)[C@@H](O)2))OC(=O)CCCCCCCCCCCCCCC)=O	InChI=1S/C49H90O15/c1-3-5-7-9-11-13-15-17-18-20-22-24-26-28-30-32-41(52)62-37(34-59-40(51)31-29-27-25-23-21-19-16-14-12-10-8-6-4-2)35-60-48-47(58)45(56)43(54)39(64-48)36-61-49-46(57)44(55)42(53)38(33-50)63-49/h17-18,37-39,42-50,53-58H,3-16,19-36H2,1-2H3/b18-17-<br><br>InChI=1S/C49H90O15/c1-3-5-7-9-11-13-15-17-18-20-21-23-25-27-29-31-40(51)59-34-37(62-41(52)32-30-28-26-24-22-19-16-14-12-10-8-6-4-2)35-60-48-47(58)45(56)43(54)39(64-48)36-61-49-46(57)44(55)42(53)38(33-50)63-49/h17-18,37-39,42-50,53-58H,3-16,19-36H2,1-2H3/b18-17-/t37-,38-,39-,42+,43+,44+,45+,46-,47-,48-,49-/m1/s1	424b	76927a	47256ab	35300ab	21583b

	1-20:2-2-18:3-monogalacto-syldiacyl-glycerol	56	804.619	18.58	Positive	***	804.5751	$C_{47}H_{80}O_{10}$	800.6283<br>745.6114<br>657.6029<br>147.0184<br>59.0198	800.6367<br>745.6188<br>657.6028<br>147.0652<br>59.0128	$C_{46}H_{88}O_{10}^{2+}$<br>$C_{43}H_{85}O_{9}^{+}$<br>$C_{40}H_{81}O_{6}^{+}$<br>$C_{6}H_{11}O_{4}^{+}$<br>$C_{2}H_{3}O_{2}^{+}$	CCCCC\C=C/C\C=C/CCCCCCCCCC(OC[C@@H](OC(=O)CCCCCCC\C=C/C\C=C/C\C=C/CC)CO[C@@H]1(O[C@H](CO)[C@H](O)[C@H](O)[C@@H](O)1))=O	InChI=1S/C47H80O10/c1-3-5-7-9-11-13-15-17-19-20-22-23-25-27-29-31-33-35-42(49)54-38-40(39-55-47-46(53)45(52)44(51)41(37-48)57-47)56-43(50)36-34-32-30-28-26-24-21-18-16-14-12-10-8-6-4-2/h6,8,11-14,17-19,21,40-41,44-48,51-53H,3-5,7,9-10,15-16,20,22-39H2,1-2H3/b8-6-,13-11-,14-12-,19-17-,21-18-/t40-,41-,44+,45+,46-,47-/m1/s1	618b	1922b	5789ab	9560a	4055ab
	Derivative of 1-20:2-2-18:3-monogalacto-syldiacyl-glycerol	57	542.391	12.28	Positive	***	***	***	471.3304<br>147.0184<br>59.0198	471.3316<br>147.0652<br>59.0128	$C_{26}H_{47}O_{7}^{+}$<br>$C_{6}H_{11}O_{4}^{+}$<br>$C_{2}H_{3}O_{2}^{+}$			112645a	99893a	103646a	115405a	93762a
**Pteroates**	(6S)-5-formyl-tetrahydro-folate tri-L-glutamate	58	732.253	16.64	Positive	***	731.2511	$C_{30}H_{37}N_{9}O_{13}$	731.2555<br>377.1557<br>377.1424	731.2511<br>377.1576<br>377.1456	$C_{30}H_{37}N_{9}O_{13}$<br>$C_{18}H_{23}N_{3}O_{6}^{2+}$<br>$C_{17}H_{21}N_{4}O_{6}^{+}$	C(NC1(\C=C/C(/C(=O)N[C@H](C(=O)[O-])CCC(N[C@H](C(=O)[O-])CCC(N[C@H](C(=O)[O-])CCC([O-])=O)=O)=O)=C\C=1))[C@H]2(CNC3(/N=C(N)\NC(=O)C(/N(C=O)2)=3))	InChI=1S/C30H37N9O13/c31-30-37-24-23(26(46)38-30)39(13-40)16(12-33-24)11-32-15-3-1-14(2-4-15)25(45)36-19(29(51)52)6-9-21(42)34-17(27(47)48)5-8-20(41)35-18(28(49)50)7-10-22(43)44/h1-4,13,16-19,32H,5-12H2,(H,34,42)(H,35,41)(H,36,45)(H,43,44)(H,47,48)(H,49,50)(H,51,52)(H4,31,33,37,38,46)/p-4/t16-,17-,18-,19-/m0/s1	9088a	10446a	14402a	16403a	9622a
		59	731.239	16.61	Positive	***	731.2511	$C_{30}H_{37}N_{9}O_{13}$						6853b	14731b	18909ab	20945ab	21331a
		60	733.230	16.62	Positive	***	731.2511	$C_{30}H_{37}N_{9}O_{13}$	732.2498<br>731.2555<br>377.1557<br>377.1424	732.2584<br>731.2511<br>377.1576<br>377.1456	$C_{30}H_{38}N_{9}O_{13}^{+}$<br>$C_{30}H_{37}N_{9}O_{13}$<br>$C_{18}H_{23}N_{3}O_{6}^{2+}$<br>$C_{17}H_{21}N_{4}O_{6}^{+}$			713b	3235b	10180a	11010a	9971a
	Derivative	61	378.109	16.58	Positive	***	***	***	377.1557<br>377.1424	377.1576<br>377.1456	$C_{18}H_{23}N_{3}O_{6}^{2+}$<br>$C_{17}H_{21}N_{4}O_{6}^{+}$			6305b	17283ab	28965a	24603a	22768a
**Phospholipids**	1-octadec-11-enoyl-sn-glycerol 3-phosphate<br><br>1-cis-vaccenoyl-sn-glycerol-3-phosphate<br><br>1-oleoyl-sn-glycerol 3-phosphate	62	435.246	17.4	Negative	***	436.2590	$C_{21}H_{41}O_{7}P$	286.2508<br>149.0009	286.2505<br>149.0034	$C_{17}H_{34}O_{3}$<br>$C_{4}H_{6}O_{4}P^{-}$	CCCCCC/C=C/CCCCCCCCCC(=O)OC[C@@H](O)COP(=O)([O-])[O-]<br><br>CCCCCC\C=C/CCCCCCCCCC(=O)OC[C@H](COP(=O)([O-])[O-])O<br><br>CCCCCCCC\C=C/CCCCCCCC(=O)OC[C@H](COP([O-])([O-])=O)O	InChI=1S/C21H41O7P/c1-2-3-4-5-6-7-8-9-10-11-12-13-14-15-16-17-21(23)27-18-20(22)19-28-29(24,25)26/h7-8,20,22H,2-6,9-19H2,1H3,(H2,24,25,26)/p-2/b8-7+/t20-/m1/s1<br>InChI=1S/C21H41O7P/c1-2-3-4-5-6-7-8-9-10-11-12-13-14-15-16-17-21(23)27-18-20(22)19-28-29(24,25)26/h7-8,20,22H,2-6,9-19H2,1H3,(H2,24,25,26)/p-2/b8-7-/t20-/m1/s1<br>InChI=1S/C21H41O7P/c1-2-3-4-5-6-7-8-9-10-11-12-13-14-15-16-17-21(23)27-18-20(22)19-28-29(24,25)26/h9-	719c	34393ab	35342ab	55098a	17958bc

											10,20,22H,2-8,11-19H2,1H3,(H2,24,25,26)/p-2/b10-9-/t20-/m1/s1					
Phosphatidy-lglyceride 18:0-22:6 derivative	63	559.306	12.52	Negative	559.306	559.3041	$C_{28}H_{48}O_9P^-$	279.2312	279.2330	$C_{18}H_{31}O_2^-$	C(CCCCCCCCCCC C)CCCCC(=O)OCC (COP(OCC(O)CO)(O)=O)OC(CCC=CC C=CCC=CCC=CCC =CCC=CCC)=O	InChI=1S/C46H79O10P/c1-3-5-7-9-11-13-15-17-19-20-21-22-24-26-28-30-32-34-36-38-46(50)56-44(42-55-57(51,52)54-40-43(48)39-47)41-53-45(49)37-35-33-31-29-27-25-23-18-16-14-12-10-8-6-4-2/h5,7,11,13,17,19,21-22,26,28,32,34,43-44,47-48H,3-4,6,8-10,12,14-16,18,20,23-25,27,29-31,33,35-42H2,1-2H3,(H,51,52)/b7-5-,13-11-,19-17-,22-21-,28-26-,34-32-	12493a	10888a	27065a	18863a	13937a
	64	504.303	12.53	Negative	504.303	504.3227	$C_{26}H_{49}O_7P^{-2}$						15766b	19055ab	19092ab	30125a	13585b
Phosphatidy-lcholine lyse (20:5)	65	540.324	12.85	Negative	***	541.3168	$C_{28}H_{48}NO_7P$	480.30312<br>85.0921<br>284.0891<br>255.2312<br>224.0675	480.3096<br>285.0977<br>284.0905<br>255.2330<br>224.0693	$C_{23}H_{47}NO_7P^-$<br>$C_9H_{20}NO_7P$<br>$C_9H_{19}NO_7P^-$<br>$C_{16}H_{31}O_2^-$<br>$C_7H_{15}NO_5P^-$	C(C(OC(CO)COP([O- 1])(=O)OCC[N+1](C)(C)C)=O)CC=CC C=CCC=CCC=CCC =CCCC	InChI=1S/C28H48NO7P/c1-5-6-7-8-9-10-11-12-13-14-15-16-17-18-19-20-21-22-28(31)36-27(25-30)26-35-37(32,33)34-24-23-29(2,3)4/h7-8,10-11,13-14,16-17,19-20,27,30H,5-6,9,12,15,18,21-26H2,1-4H3/b8-7-,11-10-,14-13-,17-16-,20-19-	10383b	11074ab	13728ab	17333a	10798ab
Phosphatidyl-choline lyso (22:6)	66	566.339	13.48	Negative	***	567.3325	$C_{30}H_{50}NO_7P$	506.3248<br>285.0921<br>281.2443	506.3252<br>285.0977<br>281.2486	$C_{25}H_{49}NO_7P^-$<br>$C_9H_{20}NO_7P$<br>$C_{18}H_{33}O_2^-$	O(CC[N+1](C)(C)C) P(OCC(OC(CCC=C CC=CCC=CCC=CC C=CCC=CCC)=O)C O)([O-1])=O	InChI=1S/C30H50NO7P/c1-5-6-7-8-9-10-11-12-13-14-15-16-17-18-19-20-21-22-23-24-30(33)38-29(27-32)28-37-39(34,35)36-26-25-31(2,3)4/h6-7,9-10,12-13,15-16,18-19,21-22,29,32H,5,8,11,14,17,20,23-28H2,1-4H3/b7-6-,10-9-,13-12-,16-15-,19-18-,22-21-	23271b	24226b	26390b	49129a	21842b
	67	566.339	13.23	Negative	***	567.3325	$C_{30}H_{50}NO_7P$						4677a	5833a	6183a	9292a	4349a
Phosphatidyl-choline lyso derivative	68	568.353	14.76	Negative	***	568.3409	$C_{30}H_{51}NO_7P^-$	506.3248<br>285.0921	506.3252<br>285.0977	$C_{25}H_{49}NO_7P^-$<br>$C_9H_{20}NO_7P$			16085a	17082a	22307a	22755a	21324a
	69	538.306	12.02	Negative	***	538.2939	$C_{28}H_{45}NO_7P^-$	538.3008<br>285.0921<br>224.0675	538.2939<br>285.0977<br>224.0693	$C_{28}H_{45}NO_7P^-$<br>$C_9H_{20}NO_7P$<br>$C_7H_{15}NO_5P^-$			5246a	5339a	4843a	10354a	4364a
1-(α-linolenoyl)-sn-glycero-3-phosphoetha-nolamine	70	476.273	12.46	Negative	***	476.2772	$C_{23}H_{43}NO_7P^+$	476.2728<br>279.2312<br>197.0436	476.2783<br>279.2330<br>197.0464	$C_{23}H_{43}NO_7P^-$<br>$C_{18}H_{31}O_2^-$<br>$C_5H_{12}NO_5P^{2-}$	CC\C=C/C\C=C/C\C =C/CCCCCCCC(OC [C@H](COP(OCC[NH3+])([O-])=O)O)=O	InChI=1S/C23H42NO7P/c1-2-3-4-5-6-7-8-9-10-11-12-13-14-15-16-17-23(26)29-20-22(25)21-31-32(27,28)30-19-18-24/h3-4,6-7,9-10,22,25H,2,5,8,11-21,24H2,1H3,(H,27,28)/b4-3-,7-6-,10-9-/t22-	88791bc	88052bc	101811b	141142a	68808c

													/m1/s1					
	1-acyl-sn-glycero-3-phosphoglycerol (n-C16/0)	71	483.265	16.28	Negative	***	484.2801	$C_{22}H_{45}O_9P$	256.2355<br>255.2312<br>228.0406<br>227.0321	256.2402<br>255.2330<br>228.0410<br>227.0337	$C_{16}H_{32}O_2$<br>$C_{16}H_{31}O_2^-$<br>$C_6H_{13}O_7P^{2-}$<br>$C_6H_{12}O_7P^{3-}$	CCCCCCCCCCCCCCCC(=O)OC[C@H](COP(=O)(OC[C@H](CO)O)[O-])O	InChI=1S/C22H45O9P/c1-2-3-4-5-6-7-8-9-10-11-12-13-14-15-22(26)29-17-21(25)19-31-32(27,28)30-18-20(24)16-23/h20-21,23-25H,2-19H2,1H3,(H,27,28)/p-1/t20-,21+/m0/s1	4745b	19297b	25638b	94753a	13780b
	1-octadec-11-enoyl-sn-glycerol 3-phosphate<br><br>1-cis-vaccenoyl-sn-glycerol-3-phosphate<br><br>1-oleoyl-sn-glycerol 3-phosphate	72	435.246	17.14	Negative	***	436.2590	$C_{21}H_{41}O_7P$	356.2865<br>282.2503<br>78.9607<br>152.9945	356.2938<br>282.25707<br>8.9606<br>152.9969	$C_{21}H_{40}O_4^{2-}$<br>$C_{18}H_{34}O_2^{2-}$<br>O_3P^{3-}<br>$C_3H_6O_5^{3-}$	CCCCCC/C=C/CCCCCCCCCC(=O)OC[C@@H](O)COP(=O)([O-])[O-]<br><br>CCCCCC\C=C/CCCCCCCCCC(=O)OC[C@H](COP(=O)([O-])[O-])O<br><br>CCCCCCCC\C=C/CCCCCCCC(=O)OC[C@H](COP([O-])([O-])=O)O	InChI=1S/C21H41O7P/c1-2-3-4-5-6-7-8-9-10-11-12-13-14-15-16-17-21(23)27-18-20(22)19-28-29(24,25)26/h7-8,20,22H,2-6,9-19H2,1H3,(H2,24,25,26)/p-2/b8-7+/t20-/m1/s1<br><br>InChI=1S/C21H41O7P/c1-2-3-4-5-6-7-8-9-10-11-12-13-14-15-16-17-21(23)27-18-20(22)19-28-29(24,25)26/h7-8,20,22H,2-6,9-19H2,1H3,(H2,24,25,26)/p-2/b8-7-/t20-/m1/s1<br><br>InChI=1S/C21H41O7P/c1-2-3-4-5-6-7-8-9-10-11-12-13-14-15-16-17-21(23)27-18-20(22)19-28-29(24,25)26/h9-10,20,22H,2-8,11-19H2,1H3,(H2,24,25,26)/p-2/b10-9-/t20-/m1/s1	753c	34393ab	35342ab	55098a	17958bc
	Derivative of phospho-lipids	73	595.283	13.98	Negative	***	***	$C_{30}H_{45}O_{10}P$	316.0501<br>279.2312<br>78.9607	316.0570<br>279.2329<br>78.9606	$C_9H_{17}O_{10}P^{2-}$<br>$C_{18}H_{31}O^{2-}$<br>O_3P^{3-}	C/C=C/C=C/C=C/C=C/C=C/C=C/C=C/C=C/CCCC(C)OC(O)COCCC(COC(COP(O)(O)=O)O)=O	-	5145b	23830ab	17876b	54985a	4062b
	1-hexadec-9-enoyl-sn-glycerol-3-phosphate	74	407.218	14.79	Negative	***	408.2277	$C_{19}H_{37}O_7P$	407.2237<br>328.2555	407.2204<br>328.2614	$C_{19}H_{36}O_7P^-$<br>$C_{19}H_{36}O_4$	CCCCCC/C=C/CCCCCCCC(=O)OC[C@@H](O)COP(=O)([O-])[O-]	InChI=1S/C19H37O7P/c1-2-3-4-5-6-7-8-9-10-11-12-13-14-15-19(21)25-16-18(20)17-26-27(22,23)24/h7-8,18,20H,2-6,9-17H2,1H3,(H2,22,23,24)/p-2/b8-7+/t18-/m1/s1	719c	11856ab	7221abc	14913a	2921bc
	Phosphatidy-linositol lyso 16:0	75	571.278	14.95	Negative	***	572.2962	$C_{25}H_{49}O_{12}P$	316.0501<br>315.0545<br>256.2355<br>255.2312<br>152.9945	316.0570<br>315.0498<br>256.2402<br>255.2330<br>152.9969	$C_9H_{17}O_{10}P^{2-}$<br>$C_9H_{16}O_{10}P^{3-}$<br>$C_{16}H_{32}O_2$<br>$C_{16}H_{31}O_2^-$<br>$C_3H_6O_5^{3-}$	CCCCCCCCCCCCCCCC(OC[C@@H](O)COP(O[C@@H]1([C@H](O)[C@H](O)[C@@H](O)[C@H](O)[C@@H](O)1)(=O)[O-])=O	InChI=1S/C25H49O12P/c1-2-3-4-5-6-7-8-9-10-11-12-13-14-15-19(27)35-16-18(26)17-36-38(33,34)37-25-23(31)21(29)20(28)22(30)24(25)32/h18,20-26,28-	2082a	8831a	13225a	14870a	13263a

													32H,2-17H2,1H3,(H,33,34)/p-1/t18-,20-,21-,22+,23-,24-,25-/m1/s1					
	Derivative of 1-palmitoyl-sn-glycerol-3-phosphate	76	409.233	16.55	Negative	***	409.2361	$C_{19}H_{38}O_7P^-$	330.2724<br>312.2677<br>256.2355<br>152.9945<br>96.9639	330.2770<br>312.2675<br>256.2402<br>152.9969<br>96.9696	$C_{19}H_{38}O_4$<br>$C_{19}H_{36}O_3^{2-}$<br>$C_{16}H_{32}O_2$<br>$C_3H_6O_5^{3-}$<br>$H_2O_4P^-$	CCCCCCCCCCCCCCCC(=O)OC[C@@H](O)COP(=O)([O-])[O-]	InChI=1S/C19H39O7P/c1-2-3-4-5-6-7-8-9-10-11-12-13-14-15-19(21)25-16-18(20)17-26-27(22,23)24/h18,20H,2-17H2,1H3,(H2,22,23,24)/p-2/t18-/m1/s1	1576b	68456a	74139a	54586ab	23001ab
	1-(9Z,12Z-Octadecadie-noyl)-2-hydroxy-sn-glycero-3-phosphoetha-nolamine	77	476.276	13.82	Negative	***	476.2783	$C_{23}H_{43}NO_7P^-$	171.0083<br>152.9945<br>96.9639	171.0064<br>152.9969<br>96.9696	$C_3H_8O_6P^-$<br>$C_3H_6O_5^{3-}$<br>$H_2O_4P^-$	CCCCCC=CCC=CCCCCCCCC(=O)OCC(COP(=O)(O)OCCN)O	InChI=1S/C23H44NO7P/c1-2-3-4-5-6-7-8-9-10-11-12-13-14-15-16-17-23(26)29-20-22(25)21-31-32(27,28)30-19-18-24/h6-7,9-10,22,25H,2-5,8,11-21,24H2,1H3,(H,27,28)/b7-6-,10-9-/t22-/m1/s1	6593b	29292b	46594ab	78225a	22163b
	Phytosphin-gosine (C18) 1-phosphate	78	397.222	16.66	Negative	***	397.2593	$C_{18}H_{40}NO_6P$	318.2616<br>300.2629<br>152.9945<br>96.9639	318.2567<br>300.2675<br>152.9969<br>96.9696	$C_{17}H_{37}NO_2P^-$<br>$C_{18}H_{36}O_3^{-2}$<br>$C_3H_6O_5^{3-}$<br>$H_2O_4P^-$	CCCCCCCCCCCCCC[C@@H](O)[C@H]([C@H](COP([O-])(=O)[O-])[NH3+])O	InChI=1S/C18H40NO6P/c1-2-3-4-5-6-7-8-9-10-11-12-13-14-17(20)18(21)16(19)15-25-26(22,23)24/h16-18,20-21H,2-15,19H2,1H3,(H2,22,23,24)/p-1/t16-,17+,18-/m0/s1	683b	7381b	16771a	7267b	5538b
	1-18:2-2-18:3-phosphatidate	79	694.468	11.60	Positive	***	694.4574	$C_{39}H_{67}O_8P$	587.3650<br>163.0901<br>95.0989	587.3707<br>163.0965<br>95.0855	$C_{31}H_{56}O_8P^+$<br>$C_7H_{15}O_4^+$<br>$C_7H_{11}^+$	CCCCC\C=C/C\C=C/CCCCCCCC(OC[C@@H](OC(=O)CCCCCCC\C=C/C\C=C/C\C=C/CC)COP([O-])(=O)[O-])=O	InChI=1S/C39H67O8P/c1-3-5-7-9-11-13-15-17-19-21-23-25-27-29-31-33-38(40)45-35-37(36-46-48(42,43)44)47-39(41)34-32-30-28-26-24-22-20-18-16-14-12-10-8-6-4-2/h6,8,11-14,17-20,37H,3-5,7,9-10,15-16,21-36H2,1-2H3,(H2,42,43,44)/p-2/b8-6-,13-11-,14-12-,19-17-,20-18-/t37-/m1/s1	1290b	8769b	17818ab	29887a	8676b
	1-18:1-2-18:3-phosphatidate	80	696.485	12.34	Positive	***	696.4730	$C_{39}H_{69}O_8P$	679.4697<br>587.3650<br>540.3539<br>163.0901<br>109.0439<br>95.0989	679.4734<br>587.3707<br>540.3580<br>163.0965<br>109.0413<br>95.0855	$C_{39}H_{68}O_7P^+$<br>$C_{31}H_{56}O_8P^+$<br>$C_{30}H_{53}O_6P$<br>$C_7H_{15}O_4^+$<br>$C_3H_{10}O_2P^+$<br>$C_7H_{11}^+$	CCCCCCCC\C=C/CCCCCCCC(OC[C@@H](OC(=O)CCCCCCC\C=C/C\C=C/C\C=C/CC)COP([O-])(=O)[O-])=O	InChI=1S/C39H69O8P/c1-3-5-7-9-11-13-15-17-19-21-23-25-27-29-31-33-38(40)45-35-37(36-46-48(42,43)44)47-39(41)34-32-30-28-26-24-22-20-18-16-14-12-10-8-6-4-2/h6,8,12,14,17-20,37H,3-5,7,9-11,13,15-16,21-36H2,1-2H3,(H2,42,43,44)/p-2/b8-6-,14-12-,19-17-,20-18-/t37-/m1/s1	1069b	20456ab	10801b	31671a	4128b
	1-acyl-sn-glycero-3-	81	424.236	8.73	Positive	***	424.2459	$C_{19}H_{39}NO_7P^+$	257.1721<br>199.1686	257.1512<br>199.1693	$C_{10}H_{26}O_5P^+$<br>$C_{12}H_{23}O_2^+$	CCCCCC/C=C/CCCCCC(OC[C@@H](	InChI=1S/C19H38NO7P/c1-2-3-4-5-6-7-8-9-10-11-	3909a	5948a	5863a	10416a	12773a

	phosphoetha-nolamine (n-C14:1)<br><br>2-acyl-sn-glycero-3-phosphoetha-nolamine (n-C14:1)								171.1368<br>145.1196	171.1380<br>145.1223	$C_{10}H_{19}O_2^+$<br>$C_8H_{17}O_2^+$	O)COP(OCC[NH3+])([O-])=O)=O<br><br>CCCCCC/C=C/CCCCCC(=O)O[C@H](CO)COP(OCC[NH3+])([O-])=O	12-13-19(22)25-16-18(21)17-27-28(23,24)26-15-14-20/h7-8,18,21H,2-6,9-17,20H2,1H3,(H,23,24)/t18-/m1/s1<br><br>InChI=1S/C19H38NO7P/c1-2-3-4-5-6-7-8-9-10-11-12-13-19(22)27-18(16-21)17-26-28(23,24)25-15-14-20/h7-8,18,21H,2-6,9-17,20H2,1H3,(H,23,24)/b8-7+/t18-/m1/s1					
	Sphingolipids (Sphingomyelin d18:1-C20:0)	82	759.653	17.57	Positive	***	759.6375	$C_{43}H_{88}N_2O_6P^+$	747.6422<br>184.1000<br>184.0646<br>18.0324	747.6375<br>184.0966<br>184.0733<br>18.0338	$C_{42}H_{88}N_2O_6P^+$<br>$C_5H_{17}N_2O_3P^{2+}$<br>$C_5H_{15}NO_4P^+$<br>NH_4^+	C(CCC)CCCCCCCCCC=CC(C(NC(=O)CCCCCCCCCCCCCCCCCCC)COP(OCC[N+1](C)(C)C)([O-1])=O)O	InChI=1S/C43H87N2O6P/c1-6-8-10-12-14-16-18-20-21-22-23-25-27-29-31-33-35-37-43(47)44-41(40-51-52(48,49)50-39-38-45(3,4)5)42(46)36-34-32-30-28-26-24-19-17-15-13-11-9-7-2/h34,36,41-42,46H,6-33,35,37-40H2,1-5H3,(H-,44,47,48,49)/b36-34+/t41-,42+/m1/s1	1489a	8921a	8782a	24048a	15776a
		83	759.631	17.75	Positive	***	759.6375	$C_{43}H_{88}N_2O_6P^+$	747.6422<br>184.1000<br>125.0303	747.6375<br>184.0966<br>125.0362	$C_{42}H_{88}N_2O_6P^+$<br>$C_5H_{17}N_2O_3P^{2+}$<br>$C_3H_{10}O_3P^+$			1922b	2462b	8169ab	12633a	13204a
	Derivative of sphingomy-elin d18:1-C20:0	84	520.408	12.29	Positive	***	***	***	184.1000<br>125.0303	184.0966<br>125.0362	$C_5H_{17}N_2O_3P^{2+}$<br>$C_3H_{10}O_3P^+$			1061497ab	877094b	1066434ab	1735373a	772368b
		85	518.781	12.53	Positive	***	***	***						3621b	3281b	5647ab	10499a	3443b
		86	532.417	12.52	Positive	***	***	***						6423a	5326a	11592a	9449a	6267a
		87	756.611	18.42	Positive	***	***	***						858b	2341b	6869b	10513b	60861a
		88	522.424	13.23	Positive	***	***	***						23692a	27609a	29343a	40339a	18989a
		89	496.406	12.84	Positive	***	***	***						44685a	41349a	50800a	64021a	41028a
		90	782.631	18.22	Positive	***	***	***						728a	4808a	22343a	53500a	37421a
		91	494.390	12.02	Positive	***	***	***						18078a	20637a	17885a	36683a	15746a
		92	516.320	11.71	Positive	***	***	***						5282a	7201a	8335a	9267a	7982a
		93	758.643	18.13	Positive	***	***	***						1620b	25582a	48302a	44707a	49826a
	Phosphatidy-lglyceride (22:6-22:6) linked with sugar	94	1081.51	6.9	Negative	***	1082.521	$C_{54}H_{83}O_{20}P$	1081.519<br>595.3708	1081.514<br>595.3769	$C_{54}H_{82}O_{20}P^-$<br>$C_{33}H_{56}O_7P^-$	C(C=CCC=CCC=CCC=CCC=CCC)C=CCCC(=O)OC(COP(O)(=O)OCC(O)CO)COC(CCC=CCC=CCC=CCC=CCC=CCC=CCC)=O	-	12599a	37125a	30190a	35496a	25346a
	Phosphatidy-lglyceride linked sugar derivative	95	638.323	12.07	Negative	638.3175	638.3231	$C_{33}H_{51}O_{10}P^{2-}$	279.2312	279.2330	$C_{18}H_{31}O_2^-$			13013b	16267b	22012b	74612a	15189b
**Acylsugars**	Sucrose 2-isovaleroyl-3-isodecanoyl-4-isobutanoate	96	669.392	17.54	Positive	***	650.3514	$C_{31}H_{54}O_{14}$	540.30261<br>33.0797<br>75.0442	540.3015<br>133.0859<br>75.0441	$C_{24}H_{46}NO_{12}^+$<br>$C_6H_{13}O_3^+$<br>$C_3H_7O_2^+$	CCC(C)C(=O)O[C@H]2([C@@H](O[C@@]1(O[C@H](CO)[C@H]([C@H](O)1)O)CO)O[C@@H]([C@@H](OC(=O)C(C)C)[C@@H]2OC(=O)CCCCCCC(C)C)CO)	InChI=1S/C31H54O14/c1-7-19(6)29(39)43-26-25(41-22(35)13-11-9-8-10-12-17(2)3)24(42-28(38)18(4)5)21(15-33)40-30(26)45-31(16-34)27(37)23(36)20(14-32)44-31/h17-21,23-27,30,32-34,36-37H,7-16H2,1-6H3/t19?,20-,21-,23-,24-,25+,26-,27+,30-	8268b	50134a	38215a	30053a	33425a
	Sucrose 2-isovaleroyl-3-isodecanoyl-4-	97	455.271	14.50	Positive	***	451.2719	$C_{31}H_{59}NO_{14}^{2+}$	415.259<br>261.2427<br>133.0797<br>433.2463	415.2406<br>261.2427<br>133.0859<br>433.2431	$C_{17}H_{37}NO_{10}^{2+}$<br>$C_{15}H_{33}O_3^+$<br>$C_6H_{13}O_3^+$<br>$C_{21}H_{37}NO_9^+$			3213a	4242a	6168a	7360a	10256a
		98	637.368	18.92	Positive	***	636.3357	$C_{30}H_{52}O_{14}$	637.3501	637.3430	$C_{30}H_{53}O_{14}^+$			4919b	27667a	20616a	15710ab	17364ab

	isobutanoate derivative	99	***	***	Positive	***	***	***	544.4005<br>478.3249<br>457.2729<br>433.2463<br>417.2798<br>387.2319	544.3964<br>478.3131<br>457.2796<br>433.2431<br>417.2847<br>387.2377	$C_{30}H_{56}O_8^{2+}$<br>$C_{24}H_{46}O_9^{2+}$<br>$C_{24}H_{41}O_8^{+}$<br>$C_{21}H_{37}O_9^{+}$<br>$C_{22}H_{41}O_7^{+}$<br>$C_{20}H_{35}O_7^{+}$		,31+/m1/s1	-	-	-	-	-

Table S2: Identification of the set of metabolites found in untreated fruits. Treatments having different lowercase letters are significantly different using one-way ANOVA and Duncan test (p ≤ 0.05)

	Compounds	Features	MS1 Feature	RT (min)	Ion mode	Monoisotopic mass			Fragments (MS/MS)			Smiles	InChI	Abundance in fruits from treated plants with chitosan (mg.mL^{-1})				
						Detected	Database	Formulae	Detected	Database	Formulae			0	0.25	0.50	0.75	1
Benzenesulfonates	4-(3-butylnonyl)-benzensulfonic acid	100	339.201	17.11	Negative	***	***	***	119.0502<br>79.9586	119.0536<br>79.9579	$C_5H_{11}OS^-$<br>O_3S^{-2}	OS(C1=CC=C(CCC(CCCCCC)CCCC)C=C1)(=O)=O	-	17618ab	21411a	6756ab	1394b	848b
	Derivative	101	311.168	14.95	Negative	***	***	***						24706ab	35491a	24741ab	6113b	655b
		102	340.199	17.17	Negative	***	***	***	120.0609	120.0687	$C_5H_{12}OS$			1147b	4788a	3363ab	612b	522b
		103	325.183	15.92	Negative	***	***	***	119.0536	119.0502	$C_5H_{11}OS^-$			8866a	4375b	2824bc	430c	305c
Benzoates	Benzoate	104	121.030	5.54	Negative	***	122.0368	$C_7H_6O_2$	121.0251<br>77.0404	121.0295<br>77.0397	$C_7H_5O_2^-$<br>$C_6H_5^-$	C(C1(\C=C/C=C\C=1))([O-])=O	InChI=1S/C7H6O2/c8-7(9)6-4-2-1-3-5-6/h1-5H,(H,8,9)/p-1	23846a	11406a	21388a	12073a	15834a
Phenylpropanoids	8-geranylumbe-lliferone	105	297.151	13.94	Negative	***	298.1569	$C_{19}H_{22}O_3$	297.1510<br>183.1403<br>114.1410<br>113.1316	297.1496<br>183.1391<br>114.1409<br>113.1336	$C_{19}H_{21}O_3^-$<br>$C_{11}H_{19}O_2^-$<br>C_8H_{18}<br>$C_8H_{17}^-$	CC(C)=CCCC(\C)=C/CC1(/C2(OC(=O)\C=C/C(\C=C/C(/O)=1)=2))	InChI=1S/C19H22O3/c1-13(2)5-4-6-14(3)7-10-16-17(20)11-8-15-9-12-18(21)22-19(15)16/h5,7-9,11-12,20H,4,6,10H2,1-3H3/b14-7+	9567ab	10753a	8458ab	2327ab	945b
	3-O-digalloyl-1,2,4,6-tetra-O-β-D-galloyl-glucose	106	1093.18	8.85	Positive	***	1092.129	$C_{48}H_{36}O_{30}$	187.0958<br>183.0640<br>129.1276	187.0965<br>183.0652<br>129.1274	$C_9H_{15}O_4^+$<br>$C_9H_{11}O_4^+$<br>$C_8H_{17}O^+$	C(OC(C1(\C=C(C(\O)=C(/C=1)\O)\O))=O)[C@H]6(O[C@H]([C@H](OC(C2(\C=C(C(\O)=C(/C=2)\O)\O))=O)[C@@H](O	InChI=1S/C48H36O30/c49-20-1-14(2-21(50)33(20)60)42(66)72-13-32-39(75-44(68)16-5-24(53)35(62)25(54)6-16)40(76-46(70)19-11-	13127a	4264b	1671b	539b	481b

	Derivative	107	175.128	5.88	Positive	***	***	***	130.0983<br>101.0893<br>74.0329	130.1026<br>101.0961<br>74.0357	$C_{7}H_{14}O_{2}^{2+}$<br>$C_{6}H_{13}O^{+}$<br>$C_{3}H_{6}O_{2}^{2+}$	C(=O)C3(\C=C(C(\O)=C(/C=3)\O)\OC(C4(\C=C(C(\O)=C(/C=4)\O)\O))=O))[C@H](OC(=O)C5(\C=C(C(\O)=C(/C=5)\O)\O))6)OC(C7(\C=C(C(\O)=C(/C=7)\O)\O))=O)	30(59)38(65)31(12-19)73-43(67)15-3-22(51)34(61)23(52)4-15)41(77-45(69)17-7-26(55)36(63)27(56)8-17)48(74-32)78-47(71)18-9-28(57)37(64)29(58)10-18/h1-12,32,39-41,48-65H,13H2/t32-,39-,40+,41-,48+/m1/s1	15688a	10910a	11487a	10604a	13598a
**Fatty acids**	Linolenate conjugated	108	447.251	15.83	Negative	***	***	***	279.2312	279.2330	$C_{18}H_{31}O_{2}^{-}$	CC\C=C/C\C=C/C\C=C/CCCCCCCC(=O)[a glycerolipid]	-	11030a	1866b	1035b	324b	133b
		109	534.465	18.41	Negative	***	***	***	255.2312<br>279.2331	255.2330<br>279.2330	$C_{16}H_{31}O_{2}^{-}$<br>$C_{18}H_{31}O_{2}^{-}$			7663a	6587ab	3794bc	4935abc	2494c
	Decanoate<br><br>4-ethyl-octanoate<br><br>8-methyl-nonanoate	110	172.146	18.54	Positive	***	172.1463	$C_{10}H_{19}O_{2}$	118.1089<br>118.0758<br>116.0783<br>117.0858	118.0994<br>118.0994<br>116.0837<br>117.0910	$C_{6}H_{14}O_{2}$<br>$C_{6}H_{14}O_{2}$<br>$C_{6}H_{12}O_{2}$<br>$C_{6}H_{12}O_{2}^{+}$	CCCCCCCCCC(=O)[O-]<br><br>CCCCC(CC)CCC([O-])=O<br><br>CC(CCCCCCC([O-])=O)C	InChI=1S/C10H20O2/c1-2-3-4-5-6-7-8-9-10(11)12/h2-9H2,1H3,(H,11,12)/p-1<br><br>InChI=1S/C10H20O2/c1-3-5-6-9(4-2)7-8-10(11)12/h9H,3-8H2,1-2H3,(H,11,12)/p-1<br><br>InChI=1S/C10H20O2/c1-9(2)7-5-3-4-6-8-10(11)12/h9H,3-8H2,1-2H3,(H,11,12)/p-1	35559a	4061b	2332b	2324b	2571b
	Derivative	111	186.256	12.13	Positive	***	***	***	173.1578<br>116.0783	173.1536<br>116.0837	$C_{10}H_{21}O_{2}^{+}$<br>$C_{6}H_{12}O_{2}$			78563a	72924ab	73111ab	71213ab	69721b
**Flavonoids**	Phlorizin	112	436.027	10.64	Negative	***	436.1369	$C_{21}H_{24}O_{10}$	229.0631<br>121.0251	229.0718<br>121.0295	$C_{10}H_{13}O_{6}^{-}$<br>$C_{7}H_{5}O^{2-}$	C(O)[C@@H]3([C@@H](O)[C@H](O)[C@@H](O)[C@H](OC2(\C(\C(CCC1(\C=C/C(/O)=C\C=1))=O)=C(/C=C(/C=2)/[O-])\O))O3)	InChI=1S/C21H24O10/c22-9-16-18(27)19(28)20(29)21(31-16)30-15-8-12(24)7-14(26)17(15)13(25)6-3-10-1-4-11(23)5-2-10/h1-2,4-5,7-8,16,18-24,26-29H,3,6,9H2/p-1/t16-,18-,19+,20-,21-/m1/s1	49182a	13295a	44026a	26821a	15101a
	Pelargonidin<br><br>Luteolindin	113	271.060	7.86	Negative	271.0612	271.0601	$C_{15}H_{11}O_{5}^{+}$	271.0600<br>270.0528<br>269.0455<br>120.0575	271.0612<br>270.0483<br>269.0418<br>120.0497	$C_{15}H_{11}O_{5}^{-}$<br>$C_{15}H_{10}O_{6}$<br>$C_{15}H_{9}O_{5}^{-}$<br>$C_{8}H_{8}O_{6}$	C3(\C(\C1(=[O+]C2(\C=C(/C=C(C(\C=C1\[O-])=2)/[O-])\O)))=C/C=C(\C=3)/O)<br><br>C3(/C=C(C(/O)=C\C(\C1(=[O+]C2(\C(/C=C\1)=C(/C=C(/C=2)/O)\O)))=3)/O)	InChI=1S/C15H10O5/c16-9-3-1-8(2-4-9)15-13(19)7-11-12(18)5-10(17)6-14(11)20-15/h1-7H,(H3-,16,17,18,19)/p-1<br><br>InChI=1S/C15H10O5/c16-9-6-12(18)10-2-4-14(20-15(10)7-9)8-1-3-11(17)13(19)5-8/h1-7H,(H3-,16,17,18,19)/p+1	86617a	52655a	64953a	54899a	29866a
	Cyanidin	114	287.056	7.07	Negative	287.0539	287.0550	$C_{15}H_{11}O_{6}^{+}$	287.0572<br>136.0478	287.0561<br>136.0524	$C_{15}H_{11}O_{6}^{-}$<br>$C_{8}H_{8}O_{2}$	C3(\C(\C1(=[O+]C2(\C=C(/C=C(C(\C=C	InChI=1S/C15H10O6/c16-8-4-11(18)9-6-	16141a	4134a	4556a	4681a	4260a

												1\[O-])=2)/[O-<br>])\O)))=C/C(/O)=C(\<br>C=3)/O)	13(20)15(21-14(9)5-8)7-<br>1-2-10(17)12(19)3-7/h1-<br>6H,(H4-<br>,16,17,18,19,20)/p-1					
	Peonidin	115	301.071	8.11	Negative	301.0753	301.0707	$C_{16}H_{13}O_6^+$	301.0768<br>286.0441<br>150.0681<br>177.0168<br>161.0253<br>137.0584	301.0718<br>286.0488<br>150.0692<br>177.0193<br>161.0244<br>137.0608	$C_{16}H_{13}O_6^-$<br>$C_{15}H_{10}O_6^{2-}$<br>$C_9H_{10}O_2^{2-}$<br>$C_9H_5O_4^-$<br>$C_9H_5O_3^-$<br>$C_8H_9O_2^-$	COC1(/C=C(\C=C/C<br>(/O)=1)/C2(C(/[O-<br>])=C\C3(\C(\[O-<br>])=C/C(/O)=C\C(\[O<br>+]=2)=3)))	InChI=1S/C16H12O6/c1-<br>21-15-4-8(2-3-<br>11(15)18)16-13(20)7-10-<br>12(19)5-9(17)6-14(10)22-<br>16/h2-7H,1H3,(H3-<br>,17,18,19,20)/p-1	41085a	2177a	46029a	9172a	50416a
	6,2'-dimetho-<br>xy-4',5'-<br>methyl-<br>enedioxy-<br>isoflavone<br>derivative	116	325.090	3.38	Negative	325.086	325.072	$C_{18}H_{13}O_6^-$	179.0370<br>135.0451	179.0350<br>135.0452	$C_9H_7O_4^-$<br>$C_8H_7O_2^-$	COC4(/C=C1(C(/OC<br>O1)=C\C(\C2(\C(C3<br>(/C=C(C(/O)=C\C(\<br>O\C=2)=3)/OC))=O)<br>)=4))	InChI=1S/C18H14O7/c1-<br>21-13-6-17-16(24-8-25-<br>17)3-9(13)11-7-23-14-5-<br>12(19)15(22-2)4-<br>10(14)18(11)20/h3-<br>7,19H,8H2,1-2H3	14401a	7310a	14626a	5704a	10476a
**Organosulfur compounds**	2-<br>mercaptobe-<br>nzothiazole	117	165.979	7.52	Negative	***	166.9863	$C_7H_5NS_2$	165.9788<br>134.0074<br>102.0410<br>31.9732	165.9791<br>134.0070<br>102.0383<br>31.9758	$C_7H_4NS_2^-$<br>$C_7H_4NS^-$<br>$C_4H_8NS^-$<br>S^{-2}			246143a	134289b	126353b	150561ab	115219b
		118	166.980	7.25	Negative	***	166.9874	$C_7H_5NS_2^{2-}$				C1(=NC2(/C=C\C=<br>C/C(/S1)=2))S	InChI=1S/C7H5NS2/c9-<br>7-8-5-3-1-2-4-6(5)10-<br>7/h1-4H,(H,8,9)	8425a	3808b	2643b	3378b	2702b
	Derivative	119	167.975	7.51	Negative	***	166.9947	$C_7H_6NS_2^-$	165.9788<br>134.0074<br>32.9820	165.9791<br>134.0070<br>32.9820	$C_7H_4NS_2^-$<br>$C_7H_4NS^-$<br>HS^-			23223a	10200b	10736b	9997b	8293b
**Terpenoids**		120	636.407	17.67	Positive	***	634.4233	$C_{40}H_{58}O_6$						11373a	12800a	6314a	6729a	4458a
	8,8-<br>dihydroxy-<br>trans-<br>neoxanthin	121	635.450	17.62	Positive	***	634.4233	$C_{40}H_{58}O_6$	242.2534<br>147.13671<br>41.0935	242.2599<br>147.1380<br>141.0910	$C_{16}H_{34}O^{2+}$<br>$C_8H_{19}O_2^+$<br>$C_8H_{13}O_2^+$	CC(/C=C/C=C(C)/C<br>=C=C1([C@@](O)(<br>C)C[C@@H](O)CC<br>(C)(C)1))=C\C=C\C<br>=C(C)\C=C\C=C(C)\<br>C(O)(O)C[C@]23(C<br>(C)(C)C[C@H](O)C<br>[C@@](C)(O2)3)	InChI=1S/C40H58O6/c1-<br>28(17-13-18-30(3)21-22-<br>34-35(5,6)23-32(41)25-<br>37(34,9)43)15-11-12-16-<br>29(2)19-14-20-<br>31(4)39(44,45)27-40-<br>36(7,8)24-33(42)26-<br>38(40,10)46-40/h11-<br>21,32-33,41-45H,23-<br>27H2,1-10H3/b12-<br>11+,17-13+,19-14+,28-<br>15+,29-16+,30-18+,31-<br>20+/t22?,32-,33-<br>,37+,38+,40-/m0/s1	9143a	7235ab	4692ab	2916b	2844b
	Oat<br>triterpenoid<br>saponin	122	1093.18	8.85	Positive	***	1093.546	$C_{55}H_{83}NO_{21}$	1023.509<br>1007.475<br>977.4956<br>116.1053	1023.516<br>1007.485<br>977.4952<br>116.1070	$C_{52}H_{79}O_{20}^+$<br>$C_{51}H_{75}O_{20}^+$<br>$C_{47}H_{77}O_{21}^+$<br>$C_6H_{14}NO^+$	C[C@]2(C=O)([C@<br>@H](OC(=O)C1(\C(<br>\NC)=C/C=C\C=1))<br>C[C@@]4(C)([C@<br>@H](C2)[C@@]35(<br>O[C@@H]3C[C@H<br>]6([C@](C)([C@](C<br>)(C[C@H](O)4)5)C<br>C[C@@H]%10([C<br>@@](C)6CC[C@H]<br>(O[C@@H]9(OC[C<br>@H](O[C@@H]7(O<br>[C@H](CO)[C@@<br>H](O)[C@H](O)[C<br>@@H](O)7))[C@H]	InChI=1S/C55H83NO21/<br>c1-49(23-59)17-32-<br>51(3,19-36(49)74-<br>45(69)25-10-8-9-11-<br>26(25)56-7)33(61)18-<br>54(6)53(5)15-12-30-<br>50(2,31(53)16-35-<br>55(32,54)77-35)14-13-<br>34(52(30,4)24-60)75-48-<br>44(76-47-<br>43(68)41(66)38(63)28(21<br>-58)72-47)39(64)29(22-<br>70-48)73-46-<br>42(67)40(65)37(62)27(20<br>-57)71-46/h8-11,23,27-	13127a	4264b	1671b	539b	481b

												(O)[C@@H](O[C@@H]8(O[C@H](CO)[C@@H](O)[C@H](O)[C@@H](O)8))9))[C@](CO)(C)%10))))	44,46-48,56-58,60-68H,12-22,24H2,1-7H3/t27-,28-,29+,30-,31-,32-,33+,34+,35-,36+,37-,38-,39+,40+,41+,42-,43-,44-,46+,47+,48+,49+,50+,51+,52+,53-,54+,55-/m1/s1					
**Phospholipids**	1-deoxy-L-glycero-tetrulose 4-phosphate<br><br>Phosphoenol-2-oxobutyrate	123	184.023	7.72	Positive	***	184.0137	$C_4H_9O_6P$	184.0117<br>167.0121<br>108.0284	184.0126<br>167.0104<br>108.0329	$C_4H_9O_6P^{2+}$<br>$C_4H_8O_5P^+$<br>$C_3H_9O_2P^{2+}$	CC(=O)[C@H](O)COP(=O)([O-])[O-]<br><br>CCC(OP([O-])([O-])=O)C([O-])=O	InChI=1S/C4H9O6P/c1-3(5)4(6)2-10-11(7,8)9/h4,6H,2H2,1H3,(H2,7,8,9)/p-2/t4-/m1/s1<br><br>InChI=1S/C4H9O6P/c1-2-3(4(5)6)10-11(7,8)9/h3H,2H2,1H3,(H,5,6)(H2,7,8,9)/p-3	25359a	18018ab	16001ab	17532ab	10623b
	Derivative	124	186.254	7.67	Positive	***	***	***						46113a	26470a	46907a	32230a	22677a
	1-stearoyl-sn-glycero-3-phosphocho-line	125	525.352	6.42	Positive	***	524.3711	$C_{26}H_{55}NO7P^+$	273.1273<br>133.1223<br>111.0147<br>272.0947	273.1330<br>133.1177<br>111.0206<br>272.0894	$C_9H_{24}NO_6P^{2+}$<br>$C_7H_{17}O_2^+$<br>$C_2H_8O_3P^+$<br>$C_8H_{19}NO_7P^+$	CCCCCCCCCCCCCCCCCC(OC[C@@H](O)COP(OCC[N+](C)(C)C)(=O)[O-])=O	InChI=1S/C26H54NO7P/c1-5-6-7-8-9-10-11-12-13-14-15-16-17-18-19-20-26(29)32-23-25(28)24-34-35(30,31)33-22-21-27(2,3)4/h25,28H,5-24H2,1-4H3/t25-/m1/s1	7292a	3062a	6199a	4892a	4975a
	2-C22:0-α,ω-dicaboxyl-lysophospha-tidic acid	126	524.319	12.78	Positive	***	524.3114	$C_{25}H_{49}O_9P^+$	209.0866<br>169.9964<br>171.0002	209.0937<br>169.9980<br>171.0053	$C_8H_{18}O_4P^+$<br>$C_3H_7O_6P$<br>$C_3H_8O_6P^+$	C(OP([O-])(=O)[O-])[C@H](OC(=O)CCCCCCCCCCCCCCCCCCCCC(=O)[O-])CO	InChI=1S/C25H49O9P/c26-21-23(22-33-35(30,31)32)34-25(29)20-18-16-14-12-10-8-6-4-2-1-3-5-7-9-11-13-15-17-19-24(27)28/h23,26H,1-22H2,(H,27,28)(H2,30,31,32)/p-3/t23-/m1/s1	7147a	7053a	5935ab	3141bc	1841c
	Derivatives of phospho-lipids	127	759.702	17.91	Positive	***	***	***	184.0966	184.1000	$C_5H_{17}N_2O_3P^{2+}$	-	-	17083a	5925b	2964bc	315c	276c
		128	758.686	18.21	Positive	***	***	***	125.0218	125.0231	$C_2H_8NO_3P^{2+}$			54429a	23702b	21322b	2960b	873b
		129	438.107	10.63	Positive	***	***	***	272.0947	272.0894	$C_8H_{19}NO_7P^+$			17177a	5958a	19187a	11229a	7375a
		130	435.324	15.29	Positive	***	***	***	98.0179	98.0122	$CH_7O_3P^{2+}$			14252a	12649a	6757a	4880a	3020a
		131	539.431	13.33	Positive	***	***	***	125.0303	125.0362	$C_3H_{10}O_3P^+$			25166a	9032b	9223b	6800b	7769b
	(trans-, trans-)-dioctadec-8-enoyl phosphati-date<br><br>1-18:0-2-	132	701.505	17.96	Positive	***	700.5043	$C_{39}H_{73}O_8P$	369.2589<br>313.1800<br>181.0579<br>125.0218<br>98.0179	369.2636<br>313.1775<br>181.0624<br>125.0231<br>98.0122	$C_{21}H_{37}O_5^+$<br>$C_{13}H_{30}O_6P^+$<br>$C_6H_{14}O_4P^+$<br>$C_2H_8NO_3P^{2+}$<br>$CH_7O_3P^{2+}$	CCCCCCCCC/C=C/CCCCCCC(=O)OC[C@@H](OC(CCCCCC/C=C/CCCCCCCCC)=O)COP([O-])(=O)[O-]<br><br>CCCCCCCCCCCCCCCCCC(OC[C@@H](OC(=O)CCCCC	InChI=1S/C39H73O8P/c1-3-5-7-9-11-13-15-17-19-21-23-25-27-29-31-33-38(40)45-35-37(36-46-48(42,43)44)47-39(41)34-32-30-28-26-24-22-20-18-16-14-12-10-8-6-4-2/h19-22,37H,3-18,23-36H2,1-2H3,(H2,42,43,44)/p-2/b21-19+,22-20+/t37-/m1/s1<br><br>InChI=1S/C39H73O8P/c1-3-5-7-9-11-13-15-17-19-21-23-25-27-29-31-	76804a	117402a	20278b	1115b	725b

	18:2-phosphatidate										CC\C=C/C\C=C/CCCCC)COP([O-])(=O)[O-])=O	33-38(40)45-35-37(36-46-48(42,43)44)47-39(41)34-32-30-28-26-24-22-20-18-16-14-12-10-8-6-4-2/h12,14,18,20,37H,3-11,13,15-17,19,21-36H2,1-2H3,(H2,42,43,44)/p-2/b14-12-,20-18-/t37-/m1/s1						
	1,2-bis-icis-vaccenoyl-phosphatidate										CCCCCC\C=C/CCCCCCCCC(OC[C@@H](OC(=O)CCCCCCCCC\C=C/CCCCCC)COP([O-])(=O)[O-])=O	InChI=1S/C39H73O8P/c1-3-5-7-9-11-13-15-17-19-21-23-25-27-29-31-33-38(40)45-35-37(36-46-48(42,43)44)47-39(41)34-32-30-28-26-24-22-20-18-16-14-12-10-8-6-4-2/h13-16,37H,3-12,17-36H2,1-2H3,(H2,42,43,44)/p-2/b15-13-,16-14-/t37-/m1/s1						
	Bisoleoyl phosphate-date										CCCCCCCC\C=C/CCCCCCCC(OC[C@@H](OC(=O)CCCCCCC\C=C/CCCCCCCC)COP([O-])(=O)[O-])=O	InChI=1S/C39H73O8P/c1-3-5-7-9-11-13-15-17-19-21-23-25-27-29-31-33-38(40)45-35-37(36-46-48(42,43)44)47-39(41)34-32-30-28-26-24-22-20-18-16-14-12-10-8-6-4-2/h17-20,37H,3-16,21-36H2,1-2H3,(H2,42,43,44)/p-2/b19-17-,20-18-/t37-/m1/s1						
